# A scalable solution for isolating human multipotent clinical-grade neural stem cells from ES precursors

**DOI:** 10.1186/s13287-019-1163-7

**Published:** 2019-03-12

**Authors:** Dasa Bohaciakova, Marian Hruska-Plochan, Rachel Tsunemoto, Wesley D. Gifford, Shawn P. Driscoll, Thomas D. Glenn, Stephanie Wu, Silvia Marsala, Michael Navarro, Takahiro Tadokoro, Stefan Juhas, Jana Juhasova, Oleksandr Platoshyn, David Piper, Vickie Sheckler, Dara Ditsworth, Samuel L. Pfaff, Martin Marsala

**Affiliations:** 1 Department of Anesthesiology, University of California San Diego School of Medicine, La Jolla, CA 92093 USA; 2 Gene Expression Laboratory, Howard Hughes Medical Institute and Salk Institute for Biological Studies, 10010 North Torrey Pines Rd, La Jolla, CA 92037 USA; 3 Department of Cellular and Molecular Medicine, University of California San Diego, La Jolla, CA 92093 USA; 4 Institute of Animal Physiology and Genetics, v.v.i., AS CR, Liběchov, Czech Republic; 5 Primary and Stem Cell Systems, Life Technologies (Thermo Fisher Scientific), 501 Charmany Drive, Madison, WI 53719 USA; 6 Sanford Stem Cell Clinical Center, University of California San Diego, La Jolla, CA 92093 USA; 7 Department of Histology and Embryology, Faculty of Medicine, Masaryk University Brno, Kamenice 3, 62500 Brno, Czech Republic; 8 Sanford Consortium for Regenerative Medicine, University of California San Diego, 2880 Torrey Pines Scenic Drive, La Jolla, CA 92037 USA

**Keywords:** Human embryonic stem cell (hESC), Neural stem cell (NSC), Spinal cord, Amyotrophic lateral sclerosis (ALS), Spinal traumatic injury, Bioinformatic tools to study xenografts

## Abstract

**Background:**

A well-characterized method has not yet been established to reproducibly, efficiently, and safely isolate large numbers of clinical-grade multipotent human neural stem cells (hNSCs) from embryonic stem cells (hESCs). Consequently, the transplantation of neurogenic/gliogenic precursors into the CNS for the purpose of cell replacement or neuroprotection in humans with injury or disease has not achieved widespread testing and implementation.

**Methods:**

Here, we establish an approach for the in vitro isolation of a highly expandable population of hNSCs using the manual selection of neural precursors based on their colony morphology (CoMo-NSC). The purity and NSC properties of established and extensively expanded CoMo-NSC were validated by expression of NSC markers (flow cytometry, mRNA sequencing), lack of pluripotent markers and by their tumorigenic/differentiation profile after in vivo spinal grafting in three different animal models, including (i) immunodeficient rats, (ii) immunosuppressed ALS rats (SOD1_G93A_), or (iii) spinally injured immunosuppressed minipigs.

**Results:**

In vitro analysis of established CoMo-NSCs showed a consistent expression of NSC markers (Sox1, Sox2, Nestin, CD24) with lack of pluripotent markers (Nanog) and stable karyotype for more than 15 passages. Gene profiling and histology revealed that spinally grafted CoMo-NSCs differentiate into neurons, astrocytes, and oligodendrocytes over a 2–6-month period in vivo without forming neoplastic derivatives or abnormal structures. Moreover, transplanted CoMo-NSCs formed neurons with synaptic contacts and glia in a variety of host environments including immunodeficient rats, immunosuppressed ALS rats (SOD1G93A), or spinally injured minipigs, indicating these cells have favorable safety and differentiation characteristics.

**Conclusions:**

These data demonstrate that manually selected CoMo-NSCs represent a safe and expandable NSC population which can effectively be used in prospective human clinical cell replacement trials for the treatment of a variety of neurodegenerative disorders, including ALS, stroke, spinal traumatic, or spinal ischemic injury.

**Electronic supplementary material:**

The online version of this article (10.1186/s13287-019-1163-7) contains supplementary material, which is available to authorized users.

## Background

Neurodegenerative diseases and traumatic CNS injuries inflict untold morbidity, mortality, and economic burden in the world [1–3]. One of the strategies considered for treating neurological dysfunction is the use of neural stem cell (NSC) transplantation to replace damaged cells and/or repopulate the tissue with cells that modulate the disease through neuroprotection [4, 5]. Although a heterochronic environment, previous animal experiments have found that the transplantation of developmentally immature neural stem cells (NSCs) into the mature CNS leads to cell expansion, migration, maturation, and functional integration of neurons, astrocytes, and oligodendrocytes into the host tissue [6–10].

Several established human NSC lines are being considered or are employed in ongoing human clinical trials for the treatment of neurodegenerative disorders, including spinal traumatic injury [11–13], ALS [14, 15], Parkinson’s disease [16–18], and stroke [19–21]. Based on their origin, NSCs can be considered in two principal categories: cells derived from immature fetal tissue that contains undifferentiated lineage-committed neural or glial precursors and NSCs derived in vitro from pluripotent precursors such as embryonic stem (ES) or induced pluripotent stem (iPS) cells. NSCs generated from fetal tissue or pluripotent cell lines have specific advantages and disadvantages with respect to the availability, expandability, and safety-tumorigenicity profile required for clinical use.

Because human fetal neural tissue-derived NSCs (FT-hNSCs) are developmentally committed to producing neurons, astrocytes, and oligodendrocytes, they have limited capacity for tumor and teratoma formation. However, there are ethical concerns and an increased risk from the inherent variability that occurs with separate isolations of FT-NSCs from different embryos. By contrast, human embryonic- and induced pluripotent stem cells have an enormous capacity for expansion. In addition, there are well defined in vitro methods for triggering NSC differentiation from ES and iPS cells [22–32]. The use of pluripotent cells as a starting source for isolating NSCs, however, carries a risk of ES contamination and therefore is a serious safety concern because of the potential for tumor or teratoma formation in cell graft recipients.

To isolate hNSCs from hESCs, previous studies have employed stringent fluorescence-activated cell sorting (FACS) or microwell adhesion schemes based on the unique cell surface profile of hNSCs (CD184^+^, CD24^+^, CD44^−^ and CD271^−^) [29]. Although FACS purification has proven to be a reliable method for isolating safe hESC-derived hNSCs in animal research studies, the cost of GMP-grade antibodies combined with the limited availability of dedicated clinical FACS instrumentation and expertise represent significant impediments for widespread adoption of hESC- and iPSC-derived hNSCs in clinical applications.

Here, we sought to identify a method for producing a reliable, uniform, and safe population of clinical grade hNSCs that was not reliant upon FACS or repeated fetal tissue derivations. We opted to use hESCs as our source material for producing hNSCs because of the scalability of ES cultures and potential for banking large stocks of well-characterized hESC and NSC lines. During neural differentiation, hESCs undergo morphogenetic events characterized by the formation of radially organized columnar epithelial cells termed neural rosettes [24, 33]. These structures comprise cells expressing early neuroectodermal markers Pax6 and Sox1 and are capable of differentiating into specific neuronal and glial cell types in response to developmental cues [33, 34]. We found that a stepwise selection process to isolate neural rosettes based on their unique morphology followed by manual picking of the emergent NSC clones was a reliable technique for hNSC isolation. To distinguish the hNSCs purified based on colony morphology from hNSC isolated using other methods, we termed them CoMo-NSCs.

Gene profiling and immunocytochemistry revealed that CoMo-NSCs lacked expression of pluripotent markers such as Nanog and expressed NSC markers Nestin, Sox1, Sox2, and CD24 through > 35 passages while maintaining a stable karyotype. To test the safety and potential applicability of CoMo-NSCs in vivo, we engrafted these cells into the spinal cord of several types of animal models: (i) naïve immunodeficient rats, (ii) pre-symptomatic ALS (SOD1^G93A^)-immunosuppressed rats, and (iii) adult immunosuppressed minipigs with chronic spinal traumatic injury. Histochemistry and comprehensive gene profiling of the engrafted cells using species-specific bioinformatic filtering revealed that CoMo-NSCs gave rise to large numbers of neurons, astrocytes, and oligodendrocytes without forming teratomas, neoplastic derivatives, or abnormal structures. Our findings indicate that CoMo-NSCs develop normally even in the context of surrounding neurodegeneration and inflammation initiated by a genetic mutation (ALS; SOD1 mutation) or spinal traumatic injury. Thus, CoMo-NSCs hold promise as a large-scale clinically relevant source of neural and glial precursors.

## Methods

### Cell culture and differentiation

Experiments were performed on three cGMP-grade cell lines of undifferentiated hESCs (H9, UCSF4, and ESI-017). hESCs were grown on gelatin-coated dishes in the presence of mouse embryonic fibroblasts (MEFs; density 24,000 cells/cm^2^). Culture media was changed every day and cells passaged every 5–7 days.

Differentiation of hESCs to NSCs was performed as described in the “Results” section entitled “Differentiation and isolation of NSCs.” Established NSCs were expanded on a cell culture dish coated with poly-l-ornithine (Sigma-Aldrich) and laminin (Thermo Fisher Scientific) (P/L) and enzymatically passaged using Accutase (Stemcell Technologies) at the seeding density of 25,000 cells/cm^2^. All media compositions and dilutions are listed in Additional file 1: Table S1.

All karyotype analyses (G-banding) were performed by Cell Line Genetics LLC (Madison, WI) from live cell cultures.

### Flow cytometry and fluorescence-activated cell sorting

FACS of NSCs was performed according to the protocol described by Yuan et al. [29] at the Human Embryonic Stem Cell Core Facility at Sanford Consortium for Regenerative Medicine (2880 Torrey Pines Scenic Dr., 92037, La Jolla, CA) using BD FACS ARIA II SORP cell sorter (BD Biosciences, Franklin Lakes, NJ, USA). After sorting of CD184^+^/CD271^−^/CD44^−^/CD24^+^ NSCs, cells were plated on P/L-coated cell culture dishes in the density 25,000/cm^2^.

Expression of extracellular and intracellular markers was determined using flow cytometry on fixed cell samples using BD LSRFortessa™ (BD Biosciences, USA). All buffer compositions are listed in Additional file 1: Table S1. All antibodies and corresponding isotype controls are listed in Additional file 2: Table S2).

### In vitro terminal neuronal and astrocyte differentiation of NSCs and indirect immunofluorescence

NSCs were plated onto glass chamber slides and induced to terminally differentiate using media supplemented with BDNF, GDNF, and cAMP (neuronal differentiation) or 10% FBS (glial differentiation) for 3–6 weeks. Detailed composition of media can be found in Additional file 1: Table S1. After induction cells were stained with neuronal and glial markers and images captured and analyzed with a Fluoview FV1000 confocal microscope (Olympus, Center Valley, PA, USA). All primary and secondary antibodies are listed in Additional file 2: Table S2.

### Electrophysiology

Whole-cell patch recordings were performed on CoMo-NSCs that were infected with HIV1-Synapsin (SYN)-green fluorescent protein (GFP) lentivirus (obtained from UCSD Vector Core, Dr. Atsushi Miyanohara, Department of Anesthesiology, UCSD) and differentiated for 5 weeks prior to recording. The recording micropipettes (tip resistance 4–6 MΩ) were filled with internal solution: 135 mM K-gluconate, 4 mM MgCl_2_, 10 mM HEPES, 10 mM EGTA, 4 mM Mg-ATP, and 0.2 mM Na-GTP (pH 7.4). Recordings were made using a MultiClamp 700B amplifier and Digidata 1440A interface (Molecular Devices). Signals were filtered at 10 kHz and sampled at 10 kHz. The whole-cell capacitance was fully compensated. The bath was constantly perfused with fresh HEPES-buffered saline: 140 mM NaCl, 5 mM KCl, 10 mM HEPES, 1 mM EGTA, 3 mM MgCl_2_, and 10 mM glucose (pH 7.4). For current-clamp recordings, cells were clamped at a range of − 60 to − 80 mV. For voltage-clamp recordings, cells were clamped at − 60 mV. Cells were visualized using an OLYMPUS BX51W1 fixed-stage upright microscope. All recordings were performed at room temperature.

### Immuno-electron microscopy

Transverse spinal cord sections (50-μm-thick) were prepared from lumbar spinal cords of immunodeficient rats at 6 months after CoMo-NSCs grafting. Sections were cut on a vibratome and cryoprotected with glycerol–dimethylsulfoxide mixture. After cryoprotection, the sections were frozen and thawed four times and treated with 1% sodium borohydride. To reduce nonspecific binding, the sections were treated with 0.3% H_2_O_2_–10% methanol in TBS (100 mM Tris-HCl and 150 mM NaCl, pH 7.6) and 3% NGS–1% bovine serum albumin in TBS. Sections were reacted overnight with mouse anti-human-specific synaptophysin (1:1000; Chemicon). Bound antibody was detected using biotinylated donkey anti-mouse IgG (1:500; GE Healthcare, Little Chalfont, UK), the ABC Elite kit (Vector Laboratories, Burlingame, CA), and diaminobenzidine (DAB) as the chromogen. After DAB detection, some sections were processed by an additional antibody labeling cycle using the same method and antibody as above. This staining strategy enhanced the signal-to-background ratio while the background labeling was kept to minimal. Immunoreacted sections were post-fixed in buffered 2% OsO_4_, rinsed and stained in 1% uranyl acetate, and then dehydrated and embedded in Epon. Ultrathin sections were contrasted with uranyl acetate and analyzed under a Zeiss EM-10 electron microscope operated at 60–80 kV. Digital electron microscopic images were processed by Adobe Photoshop CS2 (Adobe Systems).

### In vivo cell grafting, surgical procedure, and experimental groups

Experimental groups and “*n*” numbers are summarized in Additional file 3: Table S3**.**

First, the adult athymic rats (Crl:NIH-Foxn1^rnu^; Charles River) and 40-day-old immunocompetent transgenic ALS rats (SOD1^G93A^) were used for spinal NSC grafting in the rodent component of this in vivo grafting study. After cell grafting, ALS rats were continuously immunosuppressed using a combined immunosuppression protocol composed of subcutaneously implanted sustained-release tacrolimus pellet (3 mg/kg/day, continuous release) and mycophenolate mofetil (10 mg/kg/day; ip for 7 days) as previously described [35, 36]. To graft NSCs spinally, the previously described technique was used [36, 37]. Animals received 10–15 spinal NSC injections (0.5 μl each) distributed bilaterally between L2–L6 spinal segments (15,000 viable cells per injection). Cell-grafted athymic rats were sacrificed and immediately transcardially perfusion-fixed with 4% paraformaldehyde at 3 weeks, 6–8 weeks, or 6 months. SOD1^G93A^ rats survived between 56 and 70 days after grafting which corresponded with the stage of early disease onset. On the day of sacrifice, all animals were transcardially perfusion-fixed with 4% paraformaldehyde.

Second, adult minipigs with previous spinal traumatic injury were employed for spinal cell grafting. Adult female Gottingen-Minnesota minipigs (*n* = 3) were anesthetized and the Th9 spinal segment exposed after partial dorsal laminectomy of the L2–3 vertebra as previously described [38]. The exposed L3 segment was compressed (1 cm/s) with an aluminum rod (5 mm in diameter) using a computer-controlled apparatus. Compression pressure cut-off was set at 2.5 kg. After trauma, animals survived for 2.5 months before spinal NSC grafting. At 2.5 months after induction of spinal injury, animals were re-anesthetized and a chronic jugular catheter (8G) placed into the right jugular vein. The site of previous spinal cord injury was then exposed, and the dura was cut open. Animals then received a total of 20 injections of CoMo-NSCs (10 μl/injection; 20,000–30,000 cells/μl; flow rate = 2 μl/min) targeted above and below the injury epicenter. From the day of cell grafting, animals were continuously immunosuppressed by tacrolimus (0.025 mg/kg/day) for 3 months by using an externally mounted 11-day infusion pump (Baxter Infusor, USA) [39]. After survival, animals were perfusion fixed with 4% paraformaldehyde for immunofluorescence analysis of the spinal cord.

### Perfusion fixation, indirect immunofluorescence staining of spinal cord sections, and quantitative analysis of grafted cell neuronal and glial differentiation

At the end of survival, rats were anesthetized with 2 mg pentobarbital and 0.25 mg phenytoin (0.5 mL of Beuthanasia-D, Intervet/Schering-Plough Animal Health Corp., Union, NJ, USA) and transcardially perfused with 200 ml of heparinized saline followed by 250 ml of 4% paraformaldehyde (PFA) in PBS. Spinal cord sections were then prepared and stained with a combination of human-specific and non-specific antibodies **(**Additional file 2: Table S2) as previously described [35].

For quantitative analysis, sections taken from immunodeficient rats at 3 weeks, 8 weeks, and 6 months after NSC grafting were used (minimum of *n* = 4 for each time point). Three sections taken from each animal with identified grafts were used for staining and quantification. Sections were stained with hNUMA antibody in combination with neuronal and glial markers including DCX, hNSE, NeuN, hGFAP, and vimentin. The total number of double-stained grafted cells was then counted and expressed as % of the total hNUMA-stained cell population.

### RNA sequencing and data analysis

RNA was isolated from in vitro cultured NSCs or NSCs-grafted spinal cord specimens using the miRvana miRNA isolation kit (Ambion AM1560). The protocol for total RNA collection was used. Median RNA input was 740 ng (IQR 660–810 ng) and RIN scores were 9.5 ± 0.3 (as determined by Beijing Genomics Institute). Paired-end 100 bp RNA sequencing libraries were prepared using the TruSeq RNA Library Preparation Kit (v2) according to the manufacturer’s instructions (Illumina). Briefly, RNA with polyA+ tails was selected using oligo-dT beads. mRNA was then fragmented and reverse-transcribed into cDNA. cDNA was end-repaired, index adapter-ligated, and PCR amplified. AMPure XP beads (Beckman Coulter) were used to purify nucleic acids after each step. Samples were sequenced on an Illumina HiSEQ 2000 by the Beijing Genomics Institute or on an Illumina NextSeq 500 at the Salk Next Generation Sequencing Core.

TruSeq adapters were trimmed from reads. Only reads > 50 bp were retained. The remaining reads were filtered, selecting for reads with > 15 average base quality. Trimming and filtering were performed with the BBMap (BBTools) package. For gene expression quantification, we used Sailfish with Gencode’s v19 human annotation for hg19. Sailfish was run with default settings.

Because human and rat mRNA transcripts might cross-contaminate in an unpredictable manner, we tested our bioinformatics pipeline with a simulated read sorting experiment. mRNA sequencing reads from pre-transplanted NSC samples were artificially mixed with mRNA reads from a control athymic rat. The resulting mixture of mRNA reads was then processed in our pipeline to determine the rate of false positives in our species sorting method. 0.3% of rat mRNA reads falsely sorted to human (with 1.4% ambiguous), while 0.04% of human reads falsely sorted to rat (with 2.3% ambiguous). In this analysis, we mixed human and rat mRNA reads at differing proportions, up to 50% of each, and noticed that the false sorting rate remained stable at all ratios of mixing. Approximately 78% of the rat reads that falsely sorted to the human genome mapped to genes, while the remaining 22% mapped to non-exon regions of the human genome.

Differential expression testing was performed by using DESeq2, edgeR, and voom-limma in R in GLM run modes. Genes with expression levels lower than 1 count per million in all groups were discarded from final testing. *p* values were corrected for multiple comparisons within the model with the Sidak method, and genes were adjusted to control FDR with the Benjamini–Hochberg method. Final *p* values are computed from the three differential expression pipelines by taking the median Sidak/Benjamini–Hochberg corrected *p* value at each gene (i.e., significant in two of the three pipelines). Genes were considered significant at *p* < 0.05.

In order to compare gene expression between pre and post-transplantation NSCs, we first compensated for the expected error rate introduced by rat mRNA reads falsely sorted as human in the mixed-species sample of post-transplantation NSCs. To compensate for this, when quantifying gene expression from pre-transplantation NSCs, we first artificially mixed the NSCs into a background of nude rat mRNA reads at the same percentage as occurred in the actual graft tissue. We then sorted the human mRNA reads back out via the bioinformatics pipeline described. This resulted in the pre and post-transplantation NSCs having a similar percentage of false positive rat mRNA reads contaminating the sample (approximately 0.3%).

Principal component analysis was performed using a subset of genes (minimum 5 TPM in 50% of the samples). We used the “variance stabilization” transform provided by the DESeq package in R on normalized estimated counts from the Sailfish quantification pipeline prior to the analysis.

## Results

### Differentiation and isolation of NSCs

**Fig. 1:**
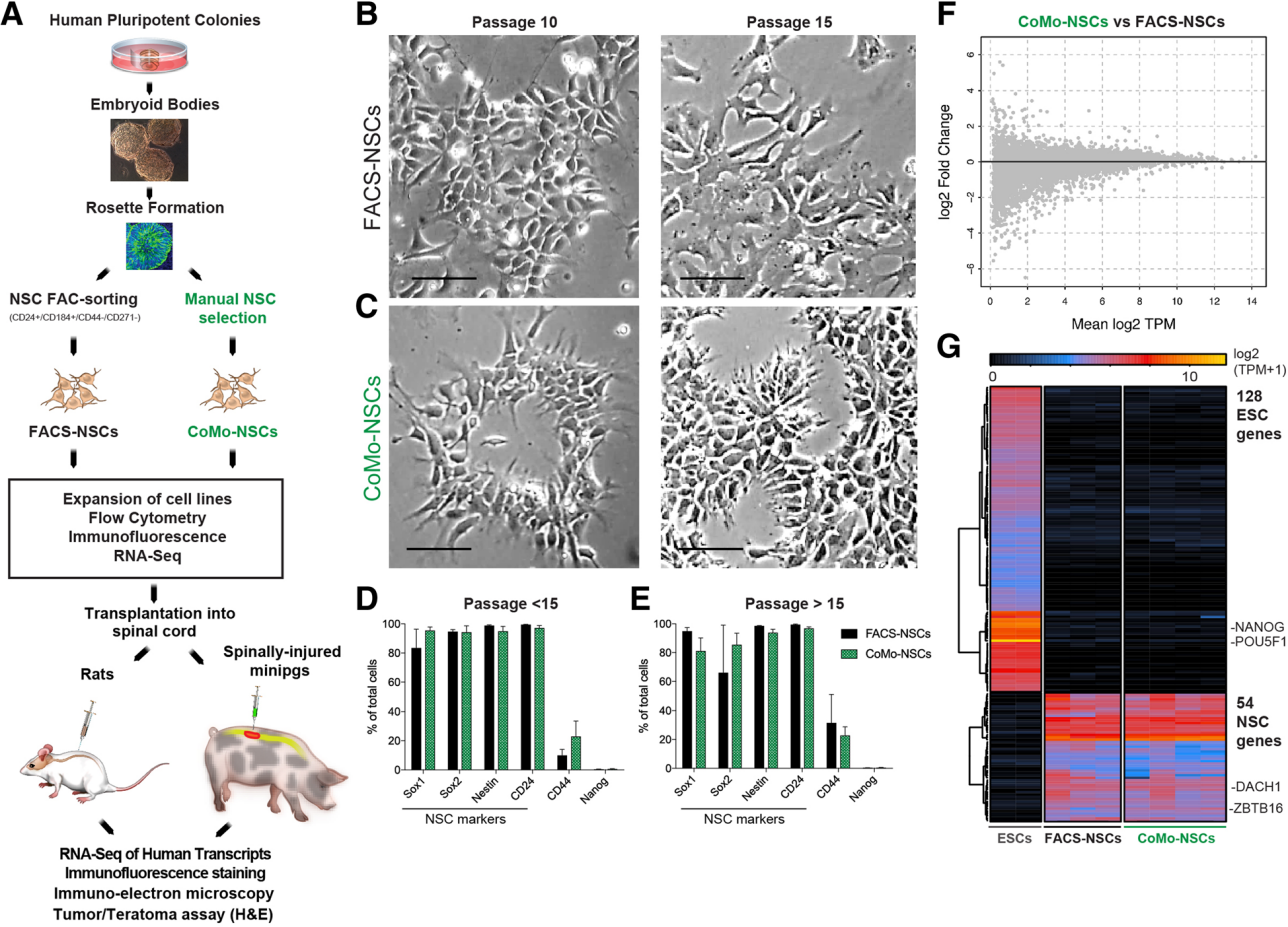
Strategy for generation, expansion, and characterization of human embryonic stem cell-derived neural stem cells. **a** Schematic diagram depicting the experimental design of in vitro ES-NSCs generation and in vitro and in vivo post-grafting characterization. **b**, **c** Morphology of NSCs derived by FACS Sorting (FACS-NSCs) and clonal morphology manual selection (CoMo-NSCs) at passage 10 and 15. **d**, **e** Expression of NSC-specific and ESC-specific markers determined by flow cytometry at passages less than 15 (**d**) and greater than 15 (**e**). Data are represented as mean ± SEM. **f** Differential gene expression plot showing the log-fold change and average transcripts per million (TPM) of each gene when comparing CoMo-NSCs and FACS-NSCs. Gray dots represent genes that are not significantly different between the two groups; the absence of black dots seen in the plot indicate that there were no genes that were significantly different between the two groups. **g** Heat map of log2(TPM+1) values of genes that distinguish ESCs from FACS-NSCs across ESC, FACS-NSC, and CoMo-NSC samples. The selection of genes is described in the methods (scale bars: **b**, **c** 50 μm)

Colonies of pluripotent hESC lines H9 (46, XX), UCSF4 (46, XX), and ESI-017 (46, XX) [40] with well-defined edges in brightfield microscopy were manually dissociated and induced to form embryoid bodies (EBs) by transferring to non-adherent dishes (Fig. 1a; Additional file 4A, B). After 4–6 days, EBs were transferred onto culture dishes coated with poly-l-ornithine and laminin (P/L) and allowed to adhere for 48 h in NSC media with 20 ng/ml of bFGF. Over a period of 4–12 days in the adherent dishes, radially organized columnar-shaped cells formed rosette structures which were readily identified in areas occupied by attached-induced EBs (Additional file 4C).

Rosettes were manually dissected and re-plated into adherent dishes, which led to the generation of secondary rosettes (R1) (Additional file 4D). Compared to the primary rosettes, R1 rosettes were smaller and remained radially organized, which distinguished them from the sparse heterogeneous epithelial cell clusters. On days 2–4, R1 rosettes were again manually picked, dissociated, and transferred to P/L-coated dishes. From these cells, islands of R2 radially organized rosettes emerged with fewer heterogeneous neuroectodermal cells apparent (Additional file 4E, F). The R2 rosette population was then used for isolating NSCs following two different isolation protocols. First, as a control, we utilized a previously established FAC-sorting isolation protocol [29]. We dissociated R2 rosettes and purified NSCs using NSC-specific surface markers, CD24^+^/CD184^+^/CD44^−^/CD271^−^ NSCs. These FAC-sorted NSCs (FACS-NSCs) were used as a control NSC population for comparison to our newly established NSC purification method, which did not employ FACS. The second purification method entailed re-plating the R2 rosettes and manually isolating the columnar epithelial cell colonies (NSCs-like cells) that appeared outside of each rosette in separate wells of 24-well plates (Additional file 4G-L). Wells that contained NSCs with a homogenous morphology, good attachment, and survival were expanded as colony morphology NSCs (CoMo-NSCs).

We noted that hESC line H9 and ESI-017 gave rise to numerous FACS-NSCs and CoMo-NSCs, whereas UCSF4 was less efficient (data not shown). This is consistent with previously noted variability in differentiation among different human ES lines [41]. Thus, selection of an appropriate hESC, and possibly iPSC, lines will likely help to improve the efficiency of NSC production from pluripotent stem cells for clinical applications regardless of the purification method. Variable differentiation characteristics are also likely to extend to different isolates of fetal precursor cells.

### Growth comparison of FACS versus morphology-based NSC isolates

To determine whether FACS- and CoMo-NSCs had similar growth characteristics, we isolated and expanded H9 and ESI-017-derived-NSCs in vitro using both purification methods and monitored their growth and differentiation characteristics. Previous reports have found that some isolates of NSCs are prone to spontaneous differentiation upon prolonged propagation [24, 42]. We found that CoMo-NSCs maintained growth rates similar to FACS-NSCs and retained their characteristic columnar morphology with refractive edges under brightfield microscopy for 10 and 15 passages (Fig. 1b, c). To determine the differentiation stage of proliferating NSCs, we used FACS to quantify the expression of a battery of cell type markers. We analyzed the expression of a pluripotency marker expressed by hES cells (Nanog), NSC-specific markers (Pax6, Sox1, Sox2, Nestin, CD24) a marker of neural crest cells (p75), and a marker of astrocytes (CD44), (Fig. 1d, e). We detected no labeling with the pluripotency marker Nanog (Fig. 1d, e) and very low levels of p75^+^ (CD271) neural crest cells and GFAP^+^ astrocytes (not shown). In contrast, both FACS- and CoMo-NSCs were highly enriched with cells expressing NSC markers. At passages < 15, CoMo-NSCs were labeled by Nestin (98.27% ± 0.53), Sox1 (88.49% ± 6.98), Sox2 (91.8% ± 2.5), and CD24 (99.1% ± 0.68). We noted some variability in Pax6 (57.26% ± 17.58) and CD44 (17.6% ± 10.08) labeling among CoMo-NSCs, but this was similar to the apparent heterogeneity of these markers within FACS-NSC cultures (Fig. 1d). This pattern of marker expression remained similar in the NSC cultures as passage number increased (Fig. 1d, e).

We next compared the gene expression between pluripotent hESC, multipotent FACS-NSCs, and CoMo-NSCs using next-generation mRNA sequencing. As expected, a large change in the mRNA reads between pluripotent versus established NSCs (both FACS-NSCs and CoMo-NSCs) was detected (Fig. 1g). In contrast, a comparison of FACS-NSCs to CoMo-NSCs grown under proliferating conditions revealed that both cultures display a nearly identical gene expression profile (Fig. 1f, g). Our findings suggest that manual selection of NSC colonies that display a radial columnar morphology with refractive cell edges is effective for enriching NSCs (i.e., CoMo-NSCs) that have a genetic profile similar to NSCs isolated by FACS purification. Because CoMo-NSCs appear to provide a distinct advantage for future GMP production and clinical applications over FACS-NSCs by circumventing the need to generate GMP-grade antibodies and the use of dedicated clinically approved sorting equipment, we further explored the properties of CoMo-NSCs.

### CoMo-NSCs efficiently self-renew and generate neurons and glia in vitro

**Fig. 2:**
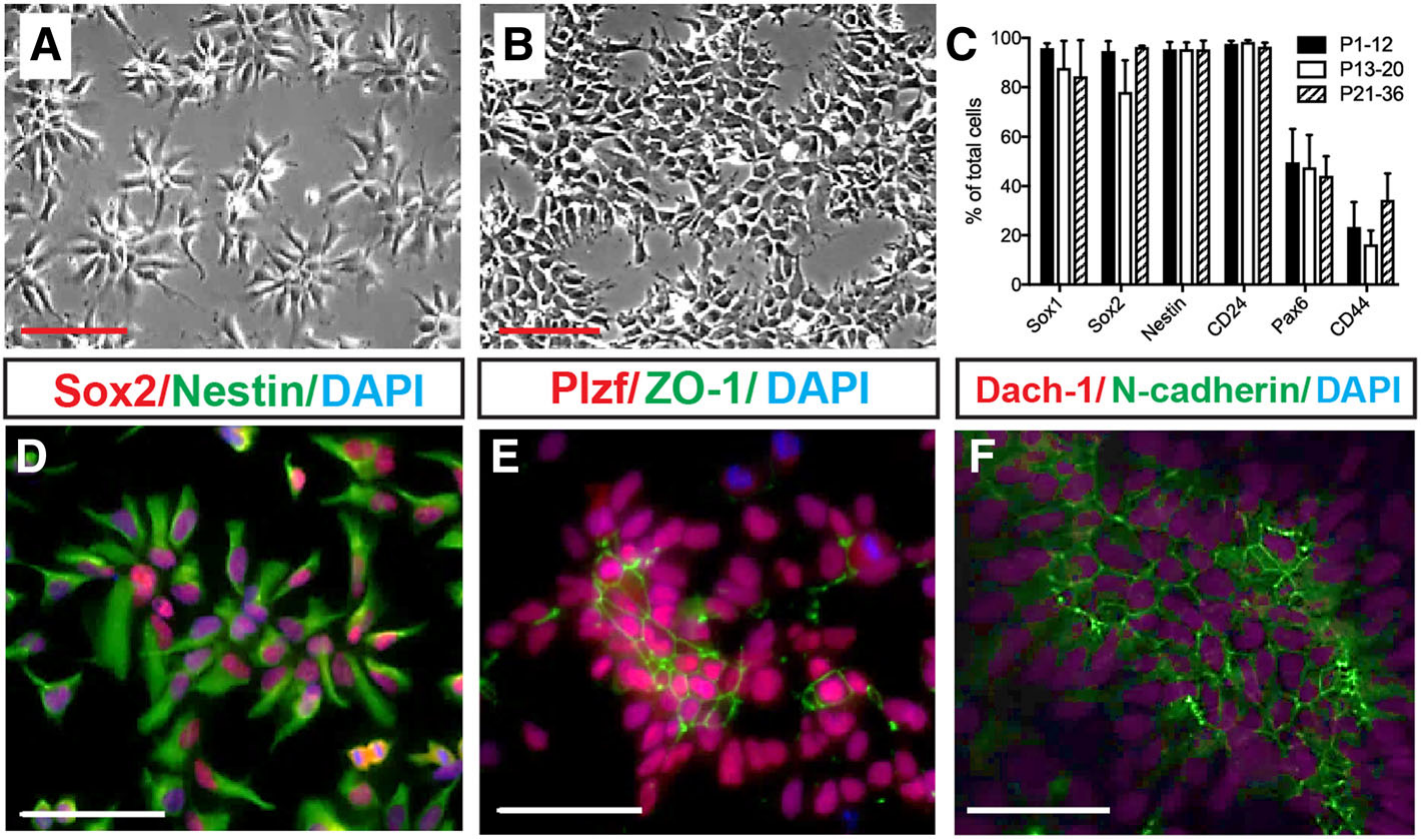
In vitro proliferating clonal morphology-derived NSCs (CoMo-NSCs) show consistent morphology and expression of markers characteristic of immature NSCs. **a**, **b** Characteristic stable morphology of CoMo-NSCs in low (**a**) and high (**b**) density. **c** Expression of selected NSC markers (Sox1, Sox2, Nestin, CD24, Pax6, and CD44) evaluated by flow cytometry at different passages. Data are represented as mean ± SEM. **d**–**f** Expression of NSC-specific markers (Sox2, Nestin, Plzf, Dach-1, N-cadherin) and tight junction protein ZO-1 in undifferentiated CoMo-NSCs as determined by indirect immunofluorescence (scale bars: **a**, **b** 25 μm; **d**–**f** 10 μm)

Based on the observation that cultured CoMo-NSCs proliferate, retain a homogenous morphology, and express NSC markers at higher passages (Fig. 1c–e), we further examined the in vitro characteristics of CoMo-NSCs from passages < 12, 13–20, and 21–36. The undifferentiated columnar morphology of CoMo-NSCs was observed for > 40 passages. Cells typically organized as clusters at both low and high density with an average doubling time of 20.96 h ± 1.51 and retained a normal karyotype (Fig. 2a, b; Additional file 5A, B). Cells expressed NSC-specific proteins Nestin, Sox2, Plzf, Dach-1, and N-cadherin at passage 17 (Fig. 2d–f). Tight junction protein ZO-1 was detected asymmetrically in the central parts of NSC clusters, confirming the polarized epithelial organization (Fig. 2e). Typical NSC markers Sox2, Sox1, Nestin, and CD24 were stably and highly expressed by CoMo-NSCs from passage < 10 to > 20, as detected using flow cytometry (Fig. 2c). As expected, astrocytic marker CD44 remained low, and Pax6 was detected at moderate levels (Fig. 2c).

**Fig. 3:**
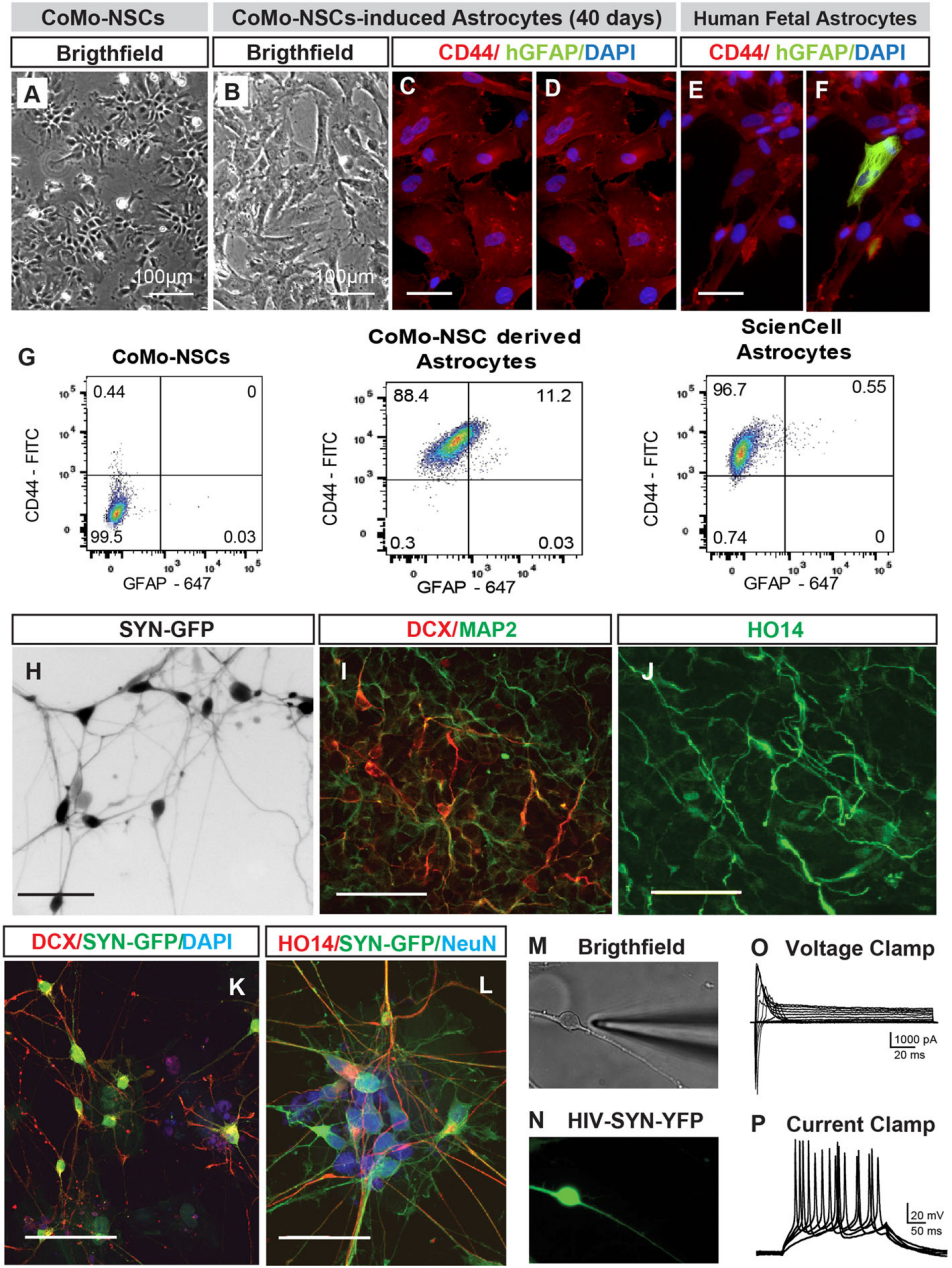
CoMo-NSCs generate astrocytes and functional neurons upon in vitro differentiation. **a**, **b** Changing morphology of differentiating CoMo-NSCs towards large flat cell type after 40 days treatment with astrocyte-inducting media. **c**–**f** Expression of human-specific GFAP (hGFAP) and CD44 in CoMo-NSCs-derived astrocytes and human fetal brain-derived astrocytes. A comparable expression pattern for both markers can be seen. **g** Representative flow cytometry plots from fixed/permeabilized cells at day 21 of astrocyte differentiation. CoMo-NSC-derived astrocytes were nearly all CD44^+^, with a fraction expressing GFAP. Primary fetal astrocytes (ScienCell) were used as a positive control, compared to CoMo-NSCs as a negative control. All cells lacked signal when analyzed in the absence of antibodies (data not shown). **h** Expression of synapsin promoter-driven GFP and appearance of neuronal morphology in CoMo-NSC-derived neurons at 6 weeks after induction using BDNF, GDNF, and cAMP. **i**–**l** Expression of neuronal markers (DCX, MAP2, human-specific axonal neurofilament HO14 and NeuN) in CoMo-NSC-derived neurons at 6 weeks after induction. **m**–**p** Patch-clamp recording in Syn-GFP neurons in vitro: voltage-clamp recording in Syn-GFP + neurons with fast inward (Na^+^) and persistent outward (K^+^) currents in depolarized membrane potentials (characteristic of neuronal cells) can be seen (**o**). In current-clamp recording (membrane potential − 65 mV), action potentials are triggered by depolarizing current pulses (**p**) (scale bars: **a**, **b** 100 μm; **c**, **e** 10 μm; **h** 200 μm; **i**–**k** 50 μm; **l** 25 μm)

A hallmark of NSCs is their ability to produce neuronal and glial (astrocytic, oligodendrocytic) progeny. We treated CoMo-NSCs with astrocyte-differentiation media (10% FBS; see the “Methods” section). Over 3–6 weeks after induction, proliferation slowed and cells exhibited a larger and flatter morphology (Fig. 3a, b). Staining with CD44 and human-specific GFAP antibodies detected high numbers of CD44+ cells, but very few or no GFAP+ cells in the CoMo-NSC culture at 3–6 weeks (Fig. 3c, d, data not shown). This staining pattern was similar to human fetal astrocyte cultures (Fig. 3e, f).

Flow cytometry confirmed that > 85% of differentiated astrocytes expressed cell surface-bound CD44, while less than 1% were GFAP+ (Fig. 3g). Next, CoMo-NSCs were cultured in neuronal differentiation media containing BDNF/GDNF/cAMP (see the “Methods” section). After induction, proliferation slowed and the morphology changed towards a neuronal phenotype with an extensive axo-dendritic arborization (Fig. 3h). These changes were accompanied with an upregulation of neuronal markers DCX, MAP2 and human-specific axonal neurofilament (HO14) (Fig. 3i, j). Very few GFAP+ astrocytes and Olig2+ oligodendrocytes were detected (not shown). To confirm that bona fide functional neurons were generated in vitro from CoMo-NSCs, we performed patch-clamp recordings on cells from an NSC clone expressing Synapsin-GFP as previously described [43]. GFP was readily detected in DCX, NeuN, and HO14+ neurons (Fig. 3k–n). Voltage clamp was used to record membrane potentials from depolarized cells and fast inward Na^+^ and persistent outward K^+^ currents characteristic of neurons were detected (Fig. 3o). In current-clamp mode, with cells at a resting membrane potential of − 65 mV, action potentials were triggered by depolarizing current pulses (Fig. 3p).

### Transplanted CoMo-NSCs differentiate into glia and neurons within the mature CNS

**Fig. 4:**
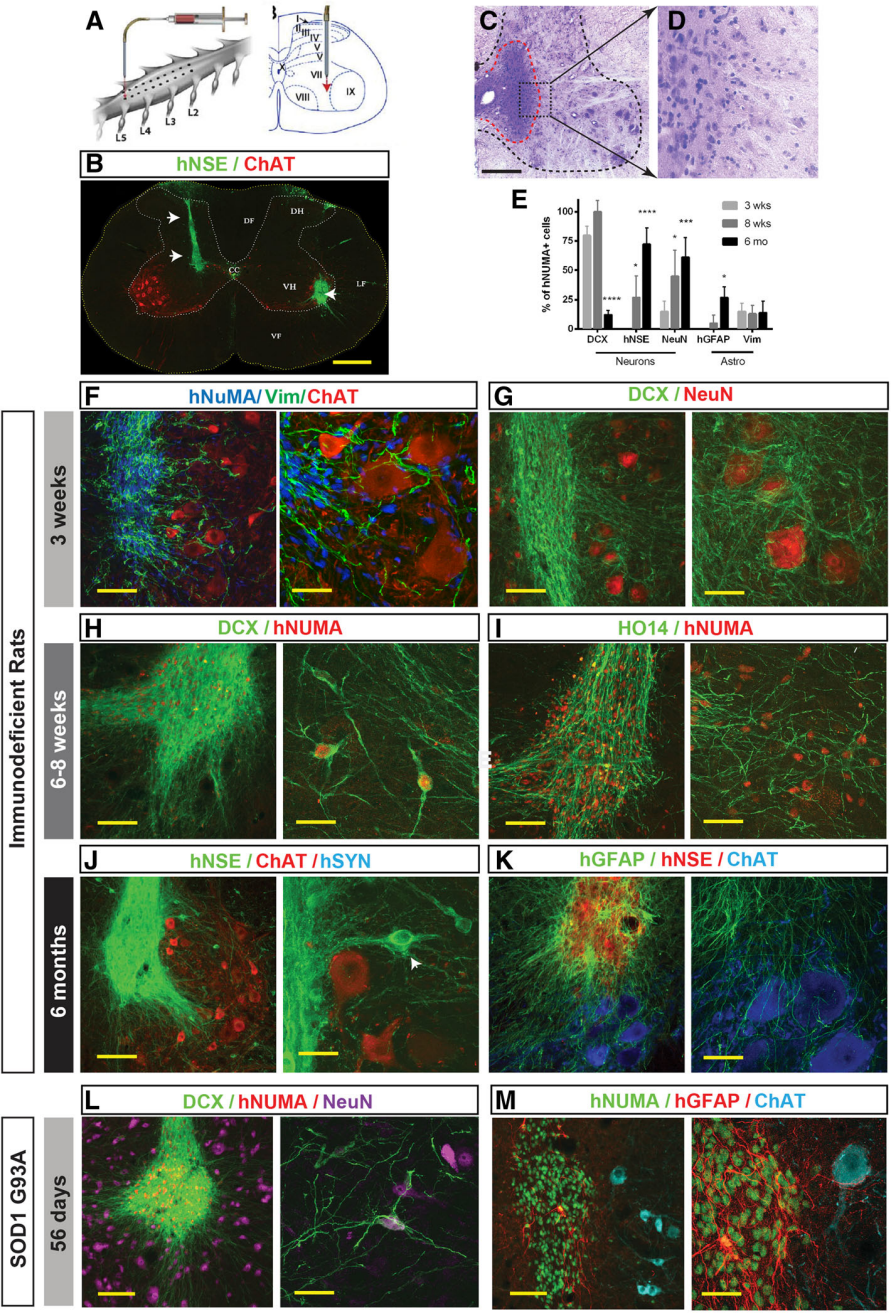
Spinally grafted clonal-derived NSCs show a long-term engraftment, no tumor formation, and time-dependent expression of human-specific markers characteristic of immature and mature neurons and glial cells. **a** Single suspension of NSCs was injected bilaterally into central gray matter of lumbar spinal cord segments in immunodeficient or G93A ALS rat using glass capillary. **b** Grafted cells were identified by expression of human-specific markers such as hNSE (green; white arrows). **c**, **d** H&E staining of lumbar spinal cord section at 6 months after NSCs grafting show well engrafted cells (red dotted area) with no detectable tumor formation. **e** Quantitative analysis of neuronal and glial differentiation at 3 weeks, 8 weeks, and 6 months after spinal NSC grafting in immunodeficient rats. Data are expressed as percent of double-stained hNUMA/DCX, hNUMA/hNSE, hNUMA/NeuN, hNUMA/GFAP, and hNUMA/Vim relative to hNUMA+ cells. Data are presented as mean ± SD. **f**, **g** At 3 weeks after grafting, a marker characteristic for proliferating immature glial precursors (Vimentin) and early post-mitotic neurons (DCX) are seen in grafted hNUMA+ cells. Extensive axo-dendritic sprouting of DCX+ positive processes surrounding the host interneurons and α-motoneurons can be seen (**g**). **h**, **i** At 6–8 weeks after NSCs transplantation, a more advanced cell migration and neuronal maturation were seen. Numerous double hNUMA/DCX+ neurons residing outside of the graft core were identified in the gray matter (**h**). Similarly, extensive axonal sprouting of HO14+ human axons was seen in the host gray matter (**i**). **j**, **k** At 6 months after NSCs grafting the appearance of mature neuronal and glial markers was identified throughout the graft. A high intensity of human-specific NSE was seen in grafted areas with several hNSE+ neurons identified outside of the graft (**j**, white arrow). Staining with human-specific GFAP antibody showed a high density of GFAP+ network with numerous hGFAP+ processes found in the ventral gray matter between α-motoneurons of the host (**k**). **l**, **m** Analysis of grafted NSCs at 56 days after grafting in G93A ALS rat lumbar spinal cord. A high density of double hNUMA/DCX-stained grafts was seen throughout the grafted segments (**l**). Staining with hGFAP showed only relatively few hGFAP+ astrocytes and these were preferentially found at the borders of individual hNUMA+ grafts (**m**) (scale bars: **b**, **c** 500 μm; **f**, **g** 100 μm; **h**, **i** 300 μm; **j** 300 μm; **k** 100 μm; **l** 300 μm; **m** 200 μm)

The signals that trigger neuroepithelial progenitor cell differentiation in vivo are normally present during embryonic development. Numerous studies with human ESC or iPSC-derived NSCs have found that they can safely differentiate into neurons, astrocytes, and oligodendrocytes when transplanted into the mature CNS in small and large animal models [10, 43, 44]. This suggests that this environment is permissive for the maturation of multipotential cells. To evaluate whether CoMo-NSCs can likewise differentiate into neurons and glia when engrafted into the mature CNS, while not forming aberrant pathological structure such as cysts, teratoma, or tumors, we grafted CoMo-NSCs into the lumbar spinal cord gray matter of athymic-immunodeficient rats (see Additional file 3: Table S3 for experimental groups; Fig. 4a). The fate of the human CoMo-NSCs was analyzed at 3 weeks, 6–8 weeks, and 6 months. All cell-grafted animals showed normal motor and sensory neurological functions with no overt signs of motor weakness, muscle spasticity or allodynia for the duration of the study (data not shown).

A histological analysis of transverse lumbar sections taken from cell-grafted segments and stained with H&E and human-specific neuron-specific enolase (hNSE) showed the engrafted cells had become incorporated into the host tissue. We did not observe hyper-cellularity due to graft over-proliferation, which can cause tissue expansion or the appearance of tumors such as teratomas and glioblastomas (Fig. 4b–d). At 3 weeks after cell grafting, immunofluorescence staining of spinal cord sections showed well-delineated hNUMA-immunoreactive grafts (Fig. 4f). To probe for the degree of neuronal and/or glial differentiation, sections were stained with early glial (Vimentin) and neuronal (DCX) markers. Numerous Vimentin+ cells were identified within individual grafts, as well as, migrating into the surrounding host tissue towards the host ChAT+ α-motor neurons (Fig. 4f). Similarly, staining with DCX (early post-mitotic neuronal marker) showed an intense DCX immunoreactivity throughout the graft with a well-developed DCX+ axo-dendritic network (Fig. 4g).

At 6–8 weeks after transplantation, in addition to an intense DCX immunoreactivity seen in hNUMA+ neurons (Fig. 4h), a high density of human axons (HO14) were observed throughout the graft region (Fig. 4i). At both 3 weeks and 6–8 weeks, minimal hGFAP immunoreactivity was detected (not shown). At 6 months post-grafting, the expression of markers which are typical of mature neural grafts (such as hNSE) was seen in the whole graft (Fig. 4j). Individual hNSE+ neurons which migrated out of the graft were also identified (Fig. 4j; white arrow). Staining with human-specific GFAP (mature astrocyte marker) and CC1 (oligodendrocyte marker) antibody at this later time point revealed a high number of mature human astrocytes and oligodendrocytes (Fig. 4k, Additional file 6A-F). Co-staining with hNUMA (human-specific nuclear marker) and Ki67 (cell division marker) antibody detected only occasional double stained cells (Additional file 6G). Quantitative analysis of early and late neuronal markers (DCX, NeuN, hNSE) and glial markers (Vimentin, hGFAP) in grafted cells showed the initial expression of early neuronal marker (DCX) and then progressive appearance of late neuronal markers (NeuN, hNSE) and mature astrocyte marker (hGFAP) at 2–6 months post-grafting (Fig. 4e).

### Transplanted CoMo-NSCs differentiate within a neurodegenerative environment

A potential application for NSCs is the treatment of neurodegenerative diseases. However, there are likely important environmental differences within the normal CNS compared to the disease state. To study the differentiation profile of CoMo-NSCs within a neurodegenerative environment, cells were grafted into lumbar spinal cord gray matter in SOD^G93A^ transgenic rats, which develop an aggressive form of amyotrophic lateral sclerosis (ALS) with a mean survival age of 100 days [45]. Animals were grafted at presymptomatic age (40 days old) and spinal cord sections analyzed using immunofluorescence staining between 56 and 70 days after grafting. Staining with hNUMA antibody showed well-delineated human cell grafts in the central gray matter (Fig. 4l). The grafts contained a high density of DCX+ neurons and some double DCX/NeuN-stained grafted neurons (Fig. 4l). Staining with hNUMA and hGFAP antibody showed a moderate density of human astrocytes in NUMA+ grafts adjacent to ChAT+ motor neurons undergoing degeneration (Fig. 4m).

### CoMo-NSC-derived neurons develop inhibitory synaptic contacts with host neurons

A key requirement to achieve a clinically relevant benefit in neuron-replacement therapies is a functional, synapse-coupled incorporation of grafted neurons into the local neuronal circuitry.

**Fig. 5:**
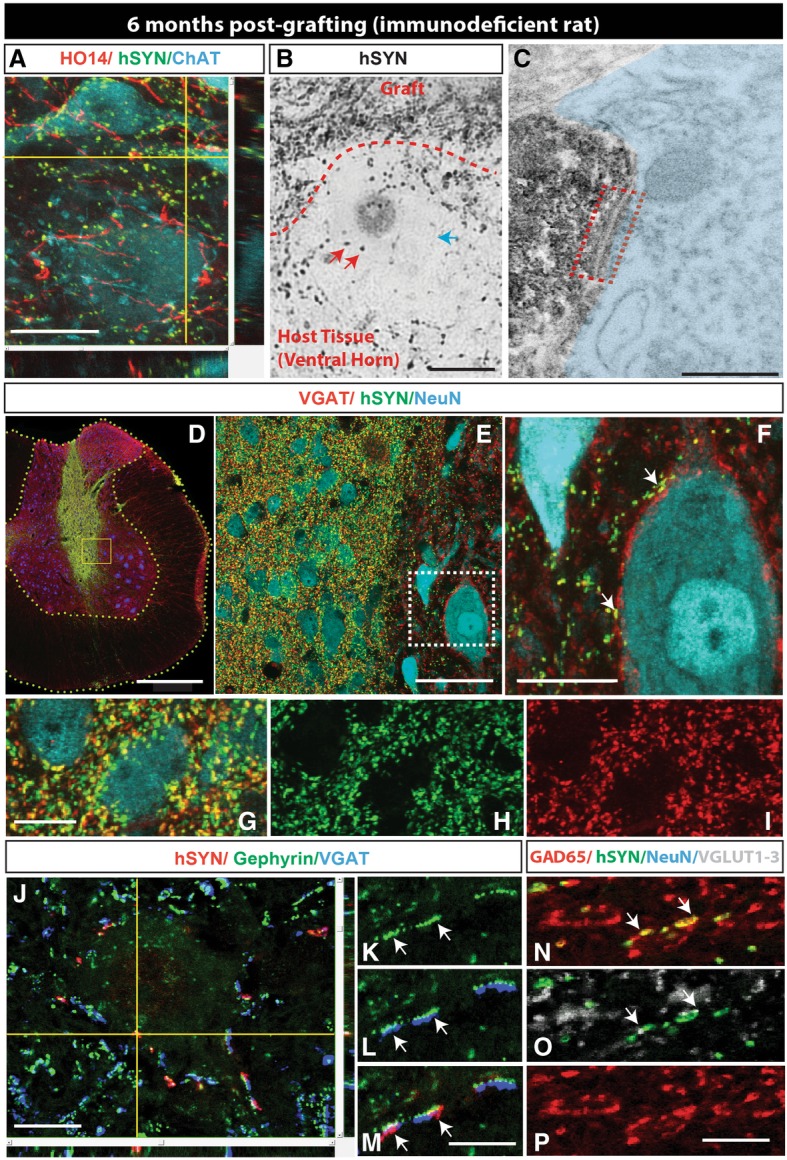
Spinally grafted clonal NSCs-derived neurons acquire preferential inhibitory neurotransmitter phenotype and develop synaptic contacts with host neurons in the immunodeficient rat at 6 months post-grafting. **a** A high density of human-specific synaptophysin puncta (hSYN) in areas occupied by human axons (HO14) and residing in the vicinity of the host ChAT+ α-motoneurons can be seen. **b**, **c** Pre-embedding immunohistochemical staining with hSYN antibody coupled with electron-optical analysis showed numerous hSYN+ puncta (**b**; semithin 1 μm section) and developed synaptic contacts between hSYN+ terminals and host neurons with readily identifiable pre- and postsynaptic densities (**c**; red boxed area). **d**–**i** Triple staining with VGAT, hSYN and NeuN antibody showed a high-density hSYN puncta through the graft as well as in surrounding host tissue. A high number of hSYN + puncta co-expressed VGAT and were residing on the membranes of the host ChAT+ α-motoneurons (**f**; white arrows). **j**–**m** Triple staining with VGAT, hSYN, and gephyrin (glycine receptor-associated protein) showed numerous double-stained hSYN/VGAT+ puncta in opposition to postsynaptically bound gephyrin+ profiles (**j**–**l**, **m**-white arrows). **n**–**p** Staining with GAD65, hSYN, NeuN, and VGLUT1–3 antibodies showed only occasional presence of VGLUT1–3+ terminals in association with hSYN puncta (scale bars: **a** 30 μm; **b** 20 μm; **c** 350 nm; **d** 500 μm; **e** 50 μm; **f** 30 μm; **g**–**i** 10 μm; **j** 20 μm; **m**, **p** 5 μm)

To study the development of synaptic contacts between grafted CoMo-NSCs-derived neurons and host neurons in more detail, sections were harvested at 6 months post-grafting in immunodeficient rats and stained with a combination of human-specific synaptophysin (hSYN), human-specific axonal neurofilament (HO14) and neurotransmitter phenotype-specific antibodies including VGAT (vesicular GABA transporter), GAD65 (glutamate decarboxylase), and VGLUT1–3 (vesicular glutamate transporters). A separate set of sections were used for pre-embedding immunohistochemistry, stained with human-specific synaptophysin antibody and then processed for electronoptical analysis of synapse formation. Immunofluorescence staining with hSYN, HO14, and ChAT antibodies showed a high density of human axons and hSYN puncta in the vicinity of host interneurons and ChAT+ α-motoneurons (Fig. 5a). Similarly, using pre-embedding immunohistochemical staining with hSYN showed numerous hSYN+ puncta in the vicinity of large host neurons (Fig. 5b). Electronoptical analysis of ultrathin sections previously stained with hSYN showed identifiable synapses between hSYN+ terminals and the host neurons with well-developed pre- and post-synaptic densities (Fig. 5c; red boxed area). Triple staining with VGAT/hSYN/NeuN antibodies revealed a high density of double-stained VGAT/hSYN puncta in the core of the graft as well as in surrounding host tissue (Fig. 5d–i). Several VGAT/hSYN+ boutons were identified on membranes of large NeuN+ neurons of the host, suggesting the development of putative inhibitory synaptic contacts (Fig. 5f; white arrows). Previous electronoptical studies have demonstrated that spatial co-localization of VGAT+ terminals with post-synaptically expressed Gephyrin corresponds with the presence of glycinergic synapses in rat spinal cord dorsal horn neurons [46]. We therefore triple-stained sections with hSYN/Gephyrin and VGAT antibodies. Numerous double hSYN/VGAT-stained terminals opposed to gephyrin immunoreactivities on neuronal membranes of the host were identified (Fig. 5j–m; white arrows). These data confirm the presence of glycinergic synapses between grafted neurons and neurons of the host. Quadruple staining with GAD65/VGLUT1–3/hSYN/NeuN antibodies revealed fewer hSYN/VGLUT1–3-stained terminals (Fig. 5n–p). Quantitative analysis showed on average 37.4 ± 2.6% of hSYN/VGAT+ terminals and 0.1 ± 0.03% of hSYN/VGLUT1–3+ terminals.

### Transcriptome analysis of transplanted CoMo-NSCs

**Fig. 6:**
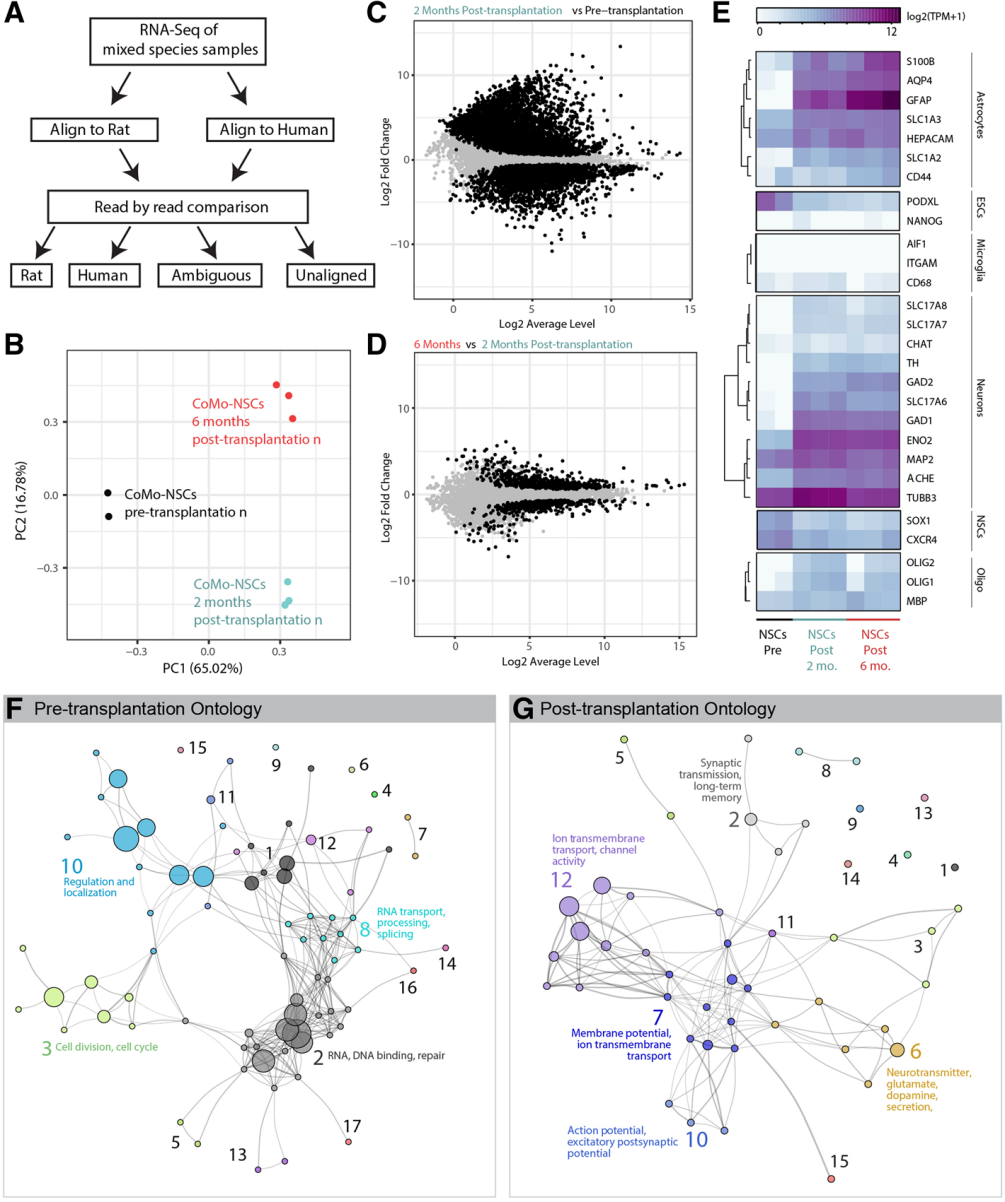
RNA-Seq analysis of transplanted CoMo-NSCs in immunodeficient rats at 2 and 6 months post-transplantation using bioinformatics-based species splitting. **a** Generalized schematic of RNA-Seq analysis pipeline using bioinformatics-based species splitting. Following analyses were conducted using the resulting human-specific transcripts only, reflecting expression profiles of the human CoMo-NSCs. **b** Principal components analysis (PCA) of three populations: CoMo-NSCs pre-transplantation (black dots, *n* = 2), CoMo-NSCs 2 months post-transplantation (red dots, *n* = 3), and CoMo-NSCs 6 months post-transplantation (blue dots, *n* = 3). The plot depicts principal components 1 (PC1) and 2 (PC2) with the percent of variance for each component. **c**, **d** Differential gene expression plot comparing CoMo-NSCs 2 months post-transplantation to CoMo-NSCs pre-transplantation (**c**) and 6 months post-transplantation to 2 months transplantation (**d**) as depicted as log2 average gene expression levels versus log2 fold change. Black dots represent genes that are significantly differentially expressed (*p* < 0.05). **e** Heat map of gene expression of canonical cell-type specific genes across the pre-transplanted and post-transplanted samples. **f** Gene ontology network of gene ontology terms overrepresented by genes enriched in the CoMo-NSCs pre-transplantation (**e**). Gene ontology groups: (1) mRNA processing, splicing, export; (2) RNA, DNA binding, repair; (3) Cell division, cell cycle; (4) Adherens junction; (5) Mismatch, double-strand break repair; (6) Ribosome biogenesis; (7) Proteoglycans and microRNAs in cancer; (8) RNA transport, processing, splicing; (9) Organ regeneration; (10) Regulation and localization; (11) Viral process; (12) Activity; (13) Assembly; (14) Gene expression; (15) Liver development; (16) ATP-dependent chromatin remodeling; (17) Translational initiation. **g** Gene ontology network of gene ontology terms overrepresented by genes enriched in the CoMo-NSCs post-transplantation (**e**). Gene ontology groups: (1) Circadian entrainment; (2) Synaptic transmission, long-term memory; (3) Signaling pathways; (4) Neuroactive ligand-receptor interaction; (5) Glutamatergic, GABAergic synapse; (6) Neurotransmitter, glutamate, dopamine secretion; (7) Membrane potential, ion transmembrane transport; (8) Morphine, nicotine addiction; (9) Locomotory behavior; (10) Action potential, excitatory postsynaptic potential; (11) Calcium ion-regulated exocytosis of neurotransmitter; (12) Ion transmembrane transport, channel activity; (13) Response to amphetamine; (14) Cardiac conduction; (15) Sensory perception of pain

Although immunostaining to detect markers of cell types is informative, we sought to identify and develop a more comprehensive method for analyzing the fate and safety profile of engrafted cells. We reasoned that mRNA sequencing could be performed on mixed-species grafts, and that human transcripts could be separated from rat transcripts through bioinformatics methods. To approach this problem, we developed a bioinformatics pipeline (Fig. 6a). mRNA was extracted from mixed-species graft tissue and was sequenced with an Illumina sequencing platform (see the “Methods” section). Every read that was generated in the sequencing run was aligned to both the rat and human genome, and alignment scores were generated based on base-pair mismatches and insertions/deletions. Reads that did not align to either genome with a threshold score of at least 90% maximum alignment were assigned as unaligned. Most reads only aligned to one, but not both genomes, and so species assignment was unambiguous. For ambiguous cases in which a read sorted to both the human and rat genomes, alignment scores between the two species were compared in order to assign the species.

We first sequenced human ESI-017 hESCs and undifferentiated CoMo-NSCs derived from this hESC line to establish baseline transcriptomes for these two cell populations. As expected, the transcriptomes of hES and CoMo-NSC cells differed significantly from one another. hESCs expressed high levels of pluripotency transcription factors including Oct4, Nanog, Sox2, Klf4 and Myc; whereas CoMo-NSCs downregulated Oct4, Nanog, Sox2, Klf4 and expressed high levels of Pax6, Sox1, Dach1, Zbtb16 (Plzf), Plagl1, and NR2F1 (Fig. 1f, data not shown). Human CoMo-NSCs derived from ESI-017 hESCs were engrafted into athymic adult rat lumbar spinal cords, and lumbar spinal cord tissue was dissected for RNA purification and sequencing 2 and 6 months after transplantation. RNA sequencing reads were assigned their species of origin using our bioinformatics pipeline, and human cell-derived transcripts were analyzed for differential gene expression and using principal component analysis (Fig. 6b–d).

This analysis revealed a large-scale shift in the transcriptome of the transplanted cells after 2 months in vivo and a further change in the RNA profile at 6 months post-grafting. At 2 and 6 months, approximately 5% of the total sequencing reads were derived from human cells, suggesting a remarkable degree of xeno-engraftment. In vitro cultured CoMo-NSCs expressed high levels of cell division genes and RNA processing factors, while 2-month engrafted CoMo-NSCs expressed axon guidance molecules, and 6-month CoMo-NSC progeny expressed higher levels of enzymes involved in metabolic processes (Fig. 6e–g, Additional file 7, Additional file 8). The 2- and 6-month CoMo-NSCs grafted cells both shared expression of synaptic vesicle and ion transport genes (Fig. 6e).

Following transplantation, NSCs are expected to generate a variety of neural cell types. We found that transcripts encoding NSC marker genes (such as Sox2 and Cxcr4) were less abundant at 2 months following engraftment and further reduced at 6 months (Fig. 6e). Concomitantly, we observed an upregulation in neuronal (Beta Tubulin class III, neuron-specific enolase, and MAP2) and astrocyte (GFAP, CD44, AQP4, S100β) genes, and to a lesser degree upregulation of oligodendrocyte factors (OLIG1, OLIG2) (Fig. 6e). Importantly, pluripotent markers of ESCs, which would represent a significant safety concern, as well as human-specific endoderm or mesoderm transcripts (indicative of teratoma formation), were not detected at any stage following CoMo-NSC engraftment.

To define the neurotransmitter identity of grafted neurons, we further examined markers of different neuronal types. We found evidence for expression of genes representative of cholinergic neurons, dopaminergic neurons, glutamatergic neurons, GABA/glycinergic inhibitory neurons, but not serotonergic neurons (Fig. 6e). Interestingly, consistent with immunofluorescence staining data, a higher activity in genes associated with neuronal synaptically mediated inhibition was seen, including GAD67 (GAD1) and GAD65 (GAD2). Similarly, only moderate expression of genes related to neuronal excitation was detected, including VGLUT1–3 (SLC17A6, SLC17A7, SLC17A8).

### CoMo-NSC engraftment in spinal injury minipigs

A transplantation protocol likely to be used in a clinical setting will employ previously well-characterized and frozen NSCs stored in clinical cell banks. In a recently completed ALS trial [47] and ongoing spinal trauma trial [11], clinical grade human fetal spinal cord-derived NSCs previously stored in liquid nitrogen (LN) were washed and shipped in hibernation buffer at 4 °C to the clinical site. After a viability test was performed (the cutoff is 70% viability), the NSCs were used directly for spinal grafting without sub-culturing. To test this likely cell preparation scenario, CoMo-NSCs were shipped frozen to our large animal facility (IAPG, Czech Republic) from UC San Diego and stored in LN for 4 weeks. On the day of grafting, the CoMo-NSCs were washed 3× in hibernation buffer and then stored at 4 °C for 2–3 h prior to being loaded into the injection device. Cells were injected just above and just below the injury epicenter (L3 spinal segment) in chronic spinally injured adult minipigs (see the “Methods” section for details). The presence of cells was studied using immunofluorescence after staining with human-specific (hNUMA, HO14, hSYN, SCI121) and non-specific (NF, VGAT, GFAP) antibodies. In all grafted animals (*n* = 3), hNUMA+ cells were detected in horizontally cut sections. Individual injection core(s) were readily identified by the presence of dense clusters of hNUMA+ cells (Additional file 9A, B). In the same areas, a dense network of human HO14+ axons was observed (Additional file 9A-C). Staining with human-specific synaptophysin (hSYN) antibody showed numerous hSYN puncta throughout the grafted region, and host neurons above and below the injury site displayed numerous hSYN terminals aligned along their surface (Additional file 9E-I). Similar as seen in spinally grafted NSCs in immunodeficient rats, co-staining with VGAT and hSYN showed numerous double-stained terminals apposed with host neurons in ventral horn and in intermediate zone (Additional file 9 J).

## Discussion

We describe a selection method to isolate expandable multipotent neural stem cells from pluripotent human embryonic stem cells. Our method relied upon serial selection of neuroepithelial cells based on their columnar morphology and radial colony organization on adherent dishes. We call the NSCs isolated using this purification method “colony morphology neural stem cells” (CoMo-NSCs) to distinguish them from NSCs purified by other previously developed methods such as FACS. The CoMo method of cell isolation offers the advantage that it markedly simplifies the number of reagents and steps associated with GMP production of large quantities of clinical grade neural stem cells. The use of cell morphology as a selection criterion for isolating neurally induced progeny from ES cells is not entirely novel for research studies [24, 27], but this approach represents a concern for clinical applications for multiple reasons including the possibility of heterogeneous mixtures of cells within the NSC culture, limited expandability, and unpredictable or unstable differentiation. Moreover, it is critical that ES-derived NSCs are not contaminated with embryonic stem cells due to their risk of tumor and/or teratoma formation.

Accordingly, the goal of our current study was threefold: First, to define the reliability of selecting NSCs by using colony morphology criteria as defined by (i) in vitro expandability, (ii) long-term stable expression of NSCs markers, (iii) lack of pluripotent markers, and (iv) ability to re-culture and expand previously frozen (i.e., banked) NSCs. Second, to characterize the engraftment properties and tumorigenic potential of NSCs transplanted into (i) the spinal cord of naïve-immunodeficient rat, (ii) the spinal cord of continuously immunosuppressed transgenic rats that develop an aggressive form of ALS (G93A), (iii) the spinal cord of continuously immunosuppressed adult minipig with chronic spinal cord injury. Third, to develop bioinformatic tools to (i) create a reference transcriptome for human clinical grade NSCs with desirable growth, differentiation, and safety characteristics and (ii) build an algorithm for deconvoluting RNA transcripts from xenografts based on species-specific SNP analysis to monitor how engrafted cells respond to their environment and detect host tissue responses to transplanted cells. Our findings indicate that colony morphology selection is effective for isolating NSCs of high purity, long-term stable self-renewal characteristics, and the ability to generate neuronal and glial progeny in the adult CNS after in vivo grafting—without detectable tumor or teratoma formation.

### Colony morphology selection is a reliable method for human NSC isolation from pluripotent ES cells

Animal studies with embryonic and induced pluripotent stem cells indicate there is great potential for cell replacement therapies [42, 43, 48–51] but the intrinsic variability among different ES and iPS cell lines combined with the risk of contaminating tumor-forming cells has slowed clinical translation**.** Isolation protocols have been developed for differentiation and purification of NSC lines from human ESC and iPSC. In general, one or more specific surface markers are used for positive/negative selection of NSCs by FACS. Depending on the developmental stage of sorted NSCs, cells can be further expanded or used directly for in vivo transplantation. This approach has shown that CD56 or the combination of CD184+/CD326− antibodies is effective for enriching neuronal precursors [51, 52], CD133, CD15 and GCTM-2 can be used to purify neurosphere-forming NSCs [53], CD133+/CD45−/CD34− cells correspond to NSCs [54], and CD24, CD15, and CD29 correspond to neuroblasts and neurons from induced hESC [55]. We have reported on a successful isolation of NSCs from induced human ES or iPS lines by using CD184^+^/CD271^−^/CD44^−^/CD24^+^ cell surface expression signature [29]. However, a limitation in applying these methods to clinical applications is the need for GMP grade antibodies. Here, we developed and validated a manual selection protocol to isolate NSCs from pluripotent hESCs that expanded > 15 passages and expressed typical NSC including Nestin, SOX1, PAX6, and SOX2. Importantly, we also verified the lack of expression of pluripotency transcription factor NANOG in established NSCs. Unlike SOX2, which plays a critical role in the maintenance of both embryonic and neural stem cells, NANOG is only expressed in undifferentiated pluripotent stem cells (reviewed in Zhang and Cui, 2014). The lack of NANOG expression thus confirms the absence of pluripotent hESCs in the established NSC population [56].

### In vivo engraftment profile and safety of CoMo-NSCs

Spinally engrafted CoMo-NSCs show a predictable time-dependent differentiation and maturation in vivo. Consistent with normal fetal development, engrafted NSCs generated neurons during the first 2 months in vivo. During the period from 2 to 6 months in vivo mature neuronal marker (NeuN, hNSE, and synaptophysin) expression increased along with glial markers (GFAP, vimentin). Using electronoptical analysis, development of synaptic contacts with the host neurons was also noted. Interestingly, confocal co-localization analysis of hSYN puncta with GAD65 and VGAT revealed that inhibitory neurons readily emerged from NSC grafts. The VGAT+ boutons derived from grafted CoMo-NSCs were spatially opposed to postsynaptic gephyrin (glycine receptor-associated protein) immunoreactivity on the host neurons. These results mirror our previous studies which observed the development of numerous inhibitory neurons from human fetal NSCs following spinal engraftment into immunosuppressed rat models of spinal ischemia [57] or spinal traumatic injury [58].

Analysis of the proliferation capacity and tumorigenesis potential of grafted cells showed dividing Ki67+ cells in graft areas with a high density of vimentin+ glial precursors, but overall cell proliferation was infrequently detected at 6 months post graft. Analysis of H&E-stained sections revealed a comparable cellularity and overall morphology of the grafts to the surrounding mature host CNS tissue with no detectable tumor formation, supporting the absence of the NANOG-positive hESC contaminants in CoMo-NSC population as identified by RNA-seq. Previous studies have demonstrated an ongoing proliferation of glial precursors with self-renewing oligodendrocyte progenitors being the main dominating proliferating cell population in the intact adult mouse spinal cord [59]. We recently reported a comparable, low-level continuing proliferation of glial precursors in the adult pig spinal cord [60]. Accordingly, we speculate that the limited mitotic activity of grafted CoMo-NSCs seen in our current study at 6 months is likely associated with ongoing oligodendrocyte proliferation and myelination at the site of human NSCs grafts.

The progressive maturation of grafted CoMo-NSCs, low-level glial-associated mitotic activity, and development of synaptic contacts with host neurons at 6 months post grafting is similar to the behavior of spinally grafted human fetal NSC line NSI-566 (Neuralstem Inc., MD, USA). This line was successfully used in 33 patients in a phase II ALS trial with no detectable side effects indicative of tumor formation after lumbar and/or cervical NSI-566 grafts in cell densities up to 16 million cells [47]. Similarly as used in previous human clinical ALS [47] or current chronic spinal trauma trial [11] which employed the NSI-566 line for spinal grafting, a combined immunosuppression protocol (tacrolimus and mycophenolate mofetil) was used in ALS rats receiving NPC grafts in our current study. The differentiation profile of grafted cells was similar compared to immunodeficient rats analyzed at approximately 2 months post grafting. These data suggest that pharmacologically induced immunosuppression does not have a major effect of on the fate and differentiation properties of grafted NPCs.

Taken together, CoMo-NSCs represent a transplantable cell population with favorable safety and differentiation characteristics.

### Transcriptomic and bioinformatic characterization of CoMo-NSCs

To establish a comprehensive reference index for the molecular features of CoMo-NSCs before and after engraftment, we performed mRNA sequencing and developed a bioinformatics method to identify human transcripts from a mixed-species graft. This method accurately identified and sorted human mRNA reads with a false positive sorting rate of only 0.3% (with 1.4% ambiguous, see methods for detail on species sorting). Gene expression was analyzed with t-distributed stochastic neighbor embedding (t-SNE) to determine the global gene expression patterns in the samples. Interestingly, no correlation among all three samples analyzed (pre-transplant CoMo-NSCs, 2 months post-transplant CoMO-NSCs and 6 months post-transplant CoMo-NSCs) was seen. Analysis of a subset of genes specific to each sample cell population showed (i) a progressive loss of immature NSCs markers (SOX1, CXR4) after grafting and (ii) the appearance of early and late glial and neuronal markers at 2 and 6 months post-grafting respectively. Analysis of CNS-specific transcripts in grafted cells showed high expression of mature neuronal markers (ENO2), neuronal inhibitory markers (GAD65, GAD67), and mature astrocyte markers (GFAP, AQP4, SLC1A3). Both the RNA sequencing data and immunofluorescence staining revealed abundant inhibitory neurons and well developed human-specific GFAP immunoreactivity. Importantly, in our current study, no expression of pluripotent markers such as NANOG or overexpression of senescence markers such as PRODH was seen in any sample. Analysis of the PODXL gene showed a progressive decrease within grafts compared to pre-transplant levels. The PODXL gene (podocalyxin-like protein) is highly expressed in pluripotent cells including proliferating NSC [61]. In addition, it was demonstrated that high PODXL expression correlates with increasing glioma grade and is a marker of poor outcome in patients with glioblastoma multiforme [62].

We demonstrate that RNA sequencing can be used to monitor the behavior of xenografts at a population level, and can serve as a sensitive tool for quantifying the expression of markers associated with unsafe growth characteristics. In addition, this technology can effectively be used to identify any alteration in post-grafting differentiation or grafted cell survival caused by in vitro expansion-induced cell(s) senescence or apoptosis [63, 64]. Accordingly, we believe that performing mRNA sequencing of pre-and post-transplant NSCs in conjunction with behavioral assessment of cell-grafted animals and post-mortem histopathological analysis of graft-targeted tissue will lead to a substantial improvement in our ability to generate and effectively screen/select safe NSCs lines to be used in a clinical setting.

## Conclusions

We have developed a new cell morphology-based selection protocol to generate an expandable population of multipotent NSCs from human embryonic stem cells. Using pre- and post-in vivo transplant analysis of NSCs, we demonstrated the phenotypic and genetic stability of in vitro long-term expanded NSCs and predictable differentiation profiles at 2 and 6 months post-spinal grafting in rats and minipigs. No tumor formation was noted. The simplicity and cost-effectiveness of this NSC selection protocol appear to provide a method of choice for the generation of clinical grade NSCs from human pluripotent (ES or iPS) cells for use in perspective clinical cell-replacement trials.

## Additional files

Additional file 1: **Table S1.** Cell culture media and buffer composition. (PDF 459 kb)

Additional file 2: **Table S2.** Antibodies used for flow cytometry, FACS, and immunofluorescence staining. (PDF 26 kb)

Additional file 3: **Table S3.** Experimental groups. (PDF 346 kb)

Additional file 4: Morphology of cell populations during the process of derivation of CoMo-NSCs from pluripotent hESCs. A—Representative image of hESC colony on mouse embryonic feeder layer. B—Manually dissociated hESCs into smaller clumps and induced to form embryoid bodies (EBs) in non-adherent cell culture conditions. C—Morphology of first neural rosettes observed at days 4–12 after plating of EBs. D—Manually separated neural rosettes, dissociated into smaller pieces and transferred to new poly-l-ornithine/laminin-coated cell culture dishes. Upon adhesion, dissected clumps of rosettes began to generate new groups of rosettes (termed “R1”). E, F—Newly enriched population of neural rosettes, both fully reformed (E) and partially reformed (F), with a very small number of contaminating cells termed as “R2”. G—Independent “clone-like populations” of NSCs visible outside of rosettes-like structures. H, I—Manually isolated single “clone-like population” of NSCs and re-plated into 24 wells plate. J, K, L—Established self-renewing population of clonal morphology NSCs, further referred to as CoMo-NSCs at low density (J), high density (K) and high magnification (L). (scale bars: A 250 μm; B, C 500 μm; D–G 250 μm; H, I 150 μm; J, K 250 μm; L 100 μm). (JPG 2540 kb)

Additional file 5: Growth curve and doubling time of CoMo-NSCs. A—Growth curve from three independent cell lines of established CoMo-NSCs. B—Average doubling time of 20.96 h (± 1.51) was calculated using formula DT = t/3.3*log b/B between day 2 and day 4 (during the exponential phase of cell growth). DT = doubling time, t = time in minutes, b = number of cells at the end time point, B = number of cells at the first time point. (JPG 247 kb)

Additional file 6: Spinally grafted clonal NSCs give rise to mature astrocyte and oligodendrocytes in the immunodeficient rat at 6 months post-grafting. A, B, C—A high-density network of human-specific GFAP+ processes in the areas of hNUMA+ human grafts can be seen. D, E, F—In the same areas a subpopulation of hNUMA+ grafted cells expressed a mature oligodendrocyte marker CC1. G—Double staining with hNUMA and Ki67 antibody showed the only occasional presence of mitotically active grafted cells. (scale bars: A 100 μm; D 80 μm; F 10 μm; G 50 μm). (JPG 4957 kb)

Additional file 7: Pre-transplantation gene ontology terms. A—Gene ontology terms overrepresented by genes enriched in the CoMo-NSCs pre-transplantation. (JPG 1072 kb)

Additional file 8: Post-transplantation gene ontology terms. A—Gene ontology terms overrepresented by genes enriched in the CoMo-NSCs post-transplantation. (JPG 902 kb)

Additional file 9: Spinally grafted CoMo-NSCs-derived neurons show a long-term engraftment, no tumor formation and extensive axonal sprouting in adult pig with previous spinal injury. A total of 20 injections of NSCs were injected bilaterally above and below spinal injury epicenter (L2–L3 segments) in chronic spinally injured adult minipigs. The presence of grafted NSCs was analyzed at 3 months after cell grafting. A, B, C—Multiple clusters of hNUMA+ grafted cells (green signal) can be identified in horizontally cut section taken from cell-grafted region. In the same areas a high density of grafted neuron-derived axons (HO14-red signal) can be seen. D, E, F, G, H, I—Staining with human-specific synaptophysin antibody (green signal) showed a high density of hSYN puncta on the host NF+ neurons. Numerous grafted neurons-derived axons (HO14; white) in the vicinity of medium-sized and large host neurons can also be seen. Only few GFAP+ grafted astrocytes (colocalizing with pan-human SCI121 immunoreactivity) were seen (E; insert). J—Triple staining with human-specific synaptophysin antibody, VGAT (vesicular GABA transporter) and NF showed numerous double hSYN/VGAT-stained puncta on the membranes of large neurons of the host (white arrows). (scale bars: A 500 μm; B 100 μm; C 50 μm; D 20 μm; E 30 μm; F 20 μm; G 10 μm; H 10 μm; I 20 μm; J 5 μm) (JPG 8408 kb)

## Abbreviations

(b)FGF: (basic) fibroblast growth factor
(c)GMP: (clinical) good manufacturing practice
(D)MEM: (Dulbecco’s) modified Eagle medium
ALS: Amyotrophic lateral sclerosis
AQP4: Aquaporin 4
ATP: Adenosine triphosphate
BDNF: Brain-derived neurotrophic factor
cAMP: Cyclic adenosine monophosphate
CHAT: Choline acetyltransferase
CNS: Central nervous system
CoMo-NSC: hNSCs purified based on colony morphology
Cxcr4: C-X-C chemokine receptor type 4
DAB: Diaminobenzidine
Dach-1: Dachshund family transcription factor 1
DCX: Doublecortin
EBs: Embryoid bodies
EGTA: Ethylene glycol-bis (β-aminoethyl ether)-*N*,*N*,*N*′,*N*′-tetraacetic acid
FACS: Fluorescence-activated cell sorting
FACS-NSCs: FAC-sorted NSCs
FT-hNSCs: Human fetal neural tissue–derived neural stem cells
GABA: Gamma-aminobutyric acid
GAD65 and 67: Glutamate decarboxylase 65 and 67
GDNF: Glial cell line-derived neurotrophic factor
GFAP: Glial fibrillary acidic protein
GFP: Green fluorescent protein
GTP: Guanosine triphosphate
H&E: Hematoxylin and eosin stain
HEPES: 4-(2-Hydroxyethyl) piperazine-1-ethanesulfonic acid, *N*-(2-hydroxyethyl) piperazine-*N*′-(2-ethanesulfonic acid)
hESCs: Human embryonic stem cells
hGFAP: Human-specific glial fibrillary acidic protein
hNSCs: Human neural stem cells
hNSE: Human-specific neuron specific enolase
hNUMA: Human-specific nuclear mitotic apparatus
HO14: Human-specific axonal neurofilament
hSYN: Human-specific synaptophysin
iPSC: Induced pluripotent stem cells
Klf4: Kruppel-like factor 4
KSR: Knockout serum replacement
LN: Liquid nitrogen
MAP2: Microtubule-associated protein 2
MEFs: Mouse embryonic fibroblasts
NeuN: Neuronal nuclei
NF: Neurofilament
NGS: Normal goat serum
NR2F1: Nuclear receptor subfamily 2 group F member 1
Oct4: POU class 5 homeobox 1
Olig1 and Olig2: Oligodendrocyte transcription factor 1 and 2
P/L: Poly-l-ornithine and laminin
Pax6: Paired box 6
PBS: Phosphate-buffered saline
PFA: Paraformaldehyde
Plagl1: PLAG1-like zinc finger 1
PODXL: Podocalyxin-like protein
S100β: S100 calcium binding protein B
SEM: Standard error of the mean
SOD1: Copper zinc superoxide dismutase 1
Sox1 and Sox2: SRY-box 1 and SRY-box 2
TBS: Tris-HCl-buffered saline
TPM: Transcripts per million
t-SNE: t-distributed stochastic neighbor embedding
VGAT: Vesicular GABA transporter
VGLUT1–3: Vesicular glutamate transporters
Zbtb16 (Plzf): Zinc finger and BTB domain containing 16
ZO-1: Zona occludens 1
