# Supplementary material for: A scalable solution for isolating human multipotent clinical-grade neural stem cells from ES precursors
Source: Stem Cell Res Ther. 2019 Mar 12;10:83. doi: 10.1186/s13287-019-1163-7 (PMC6417180; doi:10.1186/s13287-019-1163-7)
Supplement: Supplementary file 1 — Table S1. Cell culture media and buffer composition. (PDF 459 kb) [file 13287_2019_1163_MOESM1_ESM.pdf]

Table S1 Cell culture media and buffer composition

**hESC Media**		
	KO DMEM/F-12	Thermo Fisher Scientific
15%	Knockout Serum Replacement (KSR)	Thermo Fisher Scientific
2 mmol/L	L-Glutamine	Thermo Fisher Scientific
1×	MEM Non-Essential Amino Acids Solution (MEM-NEAA)	Thermo Fisher Scientific
0.5%	Penicillin-Streptomycin	Thermo Fisher Scientific
100 µmol/L	β-2 Mercaptoethanol	Sigma-Aldrich
10 ng/mL	Human Recombinant Basic Fibroblast Growth Factor-2 (bFGF)	Thermo Fisher Scientific

**EB Media**		
	KO DMEM/F-12	Thermo Fisher Scientific
15%	Knockout Serum Replacement (KSR)	Thermo Fisher Scientific
2 mmol/L	L-Glutamine	Thermo Fisher Scientific
1×	MEM Non-Essential Amino Acids Solution (MEM-NEAA)	Thermo Fisher Scientific
0.5%	Penicillin-Streptomycin	Thermo Fisher Scientific
100 µmol/L	β-2 Mercaptoethanol	Sigma-Aldrich

**NSC Media**		
	DMEM/F12	Corning
2 mmol/L	L-Glutamine	Thermo Fisher Scientific
1×	MEM Non-Essential Amino Acids Solution (MEM-NEAA)	Thermo Fisher Scientific
0.5%	Penicillin-Streptomycin	Thermo Fisher Scientific
0.5×	N2 Media Supplement	Thermo Fisher Scientific
0.5×	B27 Media Supplement	Thermo Fisher Scientific
20 ng/mL	Human Recombinant Basic Fibroblast Growth Factor-2 (bFGF)	Thermo Fisher Scientific

**Neuron Differentiation Media**		
	DMEM/F12	Thermo Fisher Scientific

2 mmol/L	L-Glutamine	Thermo Fisher Scientific
1×	MEM Non-Essential Amino Acids Solution (MEM-NEAA)	Thermo Fisher Scientific
0.5%	Penicillin-Streptomycin	Thermo Fisher Scientific
0.5×	N2 Media Supplement	Thermo Fisher Scientific
0.5×	B27 Media Supplement	Thermo Fisher Scientific
10ng/ml	Recombinant Human BDNF	PeproTech
10ng/ml	Recombinant Human GDNF	PeproTech
0.5M	cAMP	Sigma-Aldrich

**Astrocyte differentiation media**		
	DMEM-Glutamax	Thermo Fisher Scientific
10%	FBS	Thermo Fisher Scientific
0.4%	N2 Media Supplement	Thermo Fisher Scientific

**Cell Culture Coating and Dilutions**		
1:4 in Sterile Water	Poly-L-Ornithine	Sigma-Aldrich
1:200 in PBS	Laminin (1mg/ml)	Sigma-Aldrich

**FACS Buffer**		
	PBS	Thermo Fisher Scientific
2%	FBS	Thermo Fisher Scientific
1%	EDTA	Thermo Fisher Scientific

**FACS Blocking Buffer**		
	FACS Buffer	
10%	Mouse Serum	Sigma-Aldrich
