# Supplementary material for: A scalable solution for isolating human multipotent clinical-grade neural stem cells from ES precursors
Source: Stem Cell Res Ther. 2019 Mar 12;10:83. doi: 10.1186/s13287-019-1163-7 (PMC6417180; doi:10.1186/s13287-019-1163-7)
Supplement: Supplementary file 2 — Table S2. Antibodies used for flow cytometry, FACS, and immunofluorescence staining. (PDF 26 kb) [file 13287_2019_1163_MOESM2_ESM.pdf]

Table S2 Antibodies used for Flow cytometry, FACS and immunofluorescence staining.

**Antibodies used for Flow cytometry and FACS**		
Catalog #	***Name***	***Company***
555427	CD24 - FITC	BD Biosciences
555479	CD44 - PE	BD Biosciences
550538	CD44	BD Biosciences
555976	CD184 - APC	BD Biosciences
557196	CD271 - PE	BD Biosciences
560341	Nestin - APC	BD Biosciences
561470	GFAP - APC	BD Biosciences
561549	Sox1 - PerCp	BD Biosciences
561462	Pax6 - FITC	BD Biosciences
561610	Sox2 - V450	BD Biosciences
560261	Nanog - FITC	BD Biosciences

**Primary Antibodies used for Indirect immunofluorescence and Histology**		
Catalog #	***Name***	***Company***
33-9100	ZO-1	Thermo Fisher Scientific
MAB5326	Nestin	EMD Millipore
sc-22839	Plzf	Santa Cruz Biotechnology
HPA012672	Dach-1	Sigma-Aldrich
610920	N-cadherin	BD Biosciences
AB2253	Doublecortin (DCX)	EMD Millipore
C9205	GFAP (Cy3-labeled)	Sigma-Aldrich
AB5733	Vimentin	EMD Millipore
MAB2018	Sox2	R&D Systems
M4403	MAP2	Sigma-Aldrich
HO14	Human-Specific Axonal Neurofilament (HO14)	Gift from Center for Neurodegenerative Disease Research, University of Pennsylvania
AB9610	Olig2	EMD Millipore
AB15580	Ki67	Abcam
MAB377	Neuronal nuclei (NeuN)	EMD Millipore
MAB1281	Anti-Human Nuclei (hNUMA)	EMD Millipore
VPN755	human-specific neuron-specific enolase (hNSE)	Vector
MAB3786	APC	EMD Millipore
MAB332	hSYN	EMD Millipore
131002	VGAT	Synaptic Systems
AB49832	GAD65	Abcam

AB5905	VGLUT1	EMD Millipore
AB2251	VGLUT2	EMD Millipore
AB5421	VGLUT3	EMD Millipore
AB144P	ChAT	Chemicon

**Fluorescent-conjugated secondary donkey antibodies**		
Dilution	***Name***	***Company***
1:500	Alexa® Fluor 488	Jackson Immuno Research
1:500	Alexa® Fluor 647	Jackson Immuno Research
1:500	Alexa® Fluor 555	Thermo Fisher Scientific
