# Supplementary material for: A scalable solution for isolating human multipotent clinical-grade neural stem cells from ES precursors
Source: Stem Cell Res Ther. 2019 Mar 12;10:83. doi: 10.1186/s13287-019-1163-7 (PMC6417180; doi:10.1186/s13287-019-1163-7)
Supplement: Supplementary file 3 — Table S3. Experimental groups. (PDF 346 kb) [file 13287_2019_1163_MOESM3_ESM.pdf]

Table S3 Experimental Groups.

Post cell-grafting survival/assays	3 weeks IF	6-8 weeks / 3 months(*) IF/ mRNA seq	6 months IF/ iEM/ mRNA seq
**Athymic rats**	**n=12**	**n=6 / n=3**	**n=6 / n=3 / n=3**
**G93A ALS rats**	**--**	**n=8**	**--**
**Adult pigs**	**--**	**n=3 (*)**	**--**

IF: immunofluorescence, **mRNA seq**: mRNA sequencing, **iEM**: immune-electron microscopy
