# Supplementary figures and images for: A scalable solution for isolating human multipotent clinical-grade neural stem cells from ES precursors
Source: Stem Cell Res Ther. 2019 Mar 12;10:83. doi: 10.1186/s13287-019-1163-7 (PMC6417180; doi:10.1186/s13287-019-1163-7)

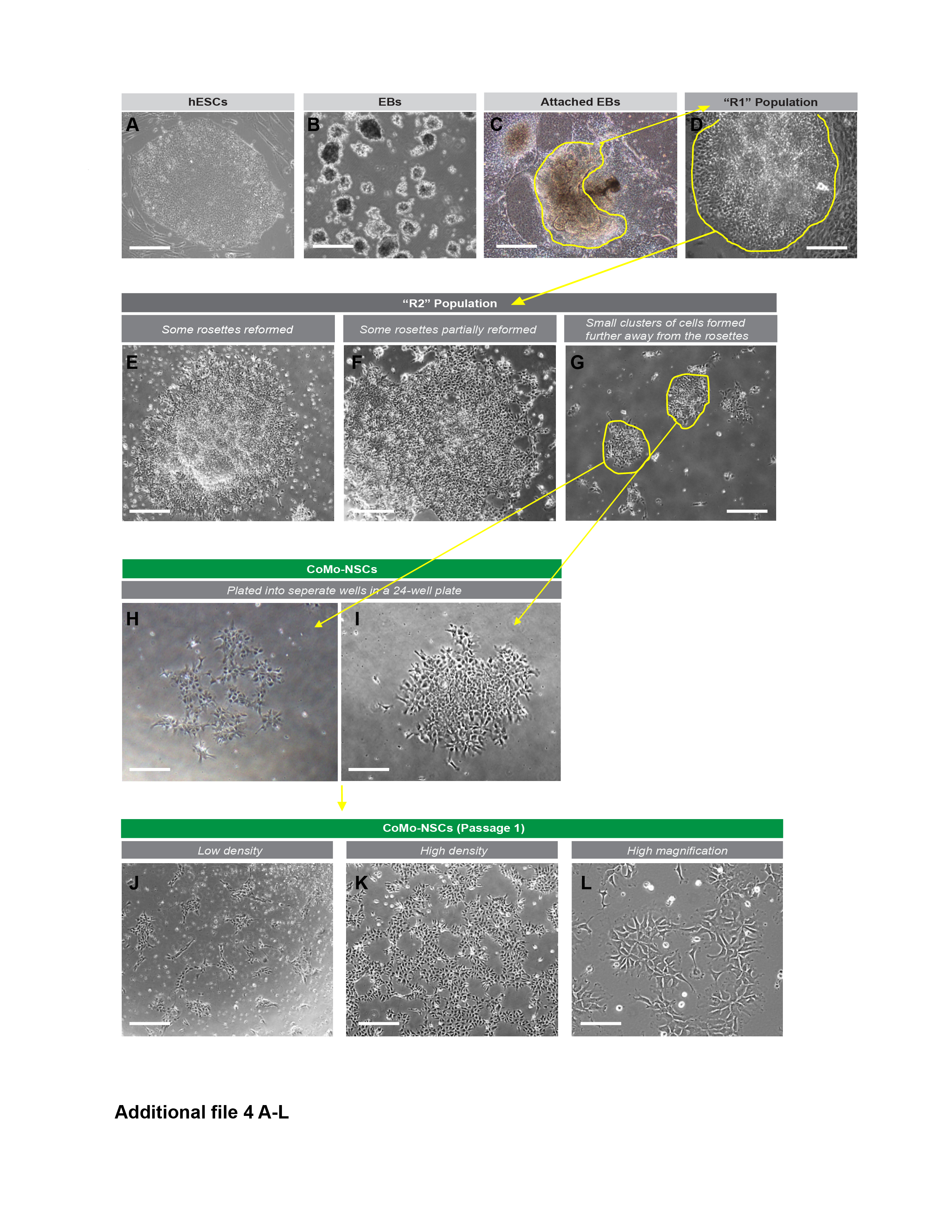

Supplement: Supplementary file 4 — Morphology of cell populations during the process of derivation of CoMo-NSCs from pluripotent hESCs. A—Representative image of hESC colony on mouse embryonic feeder layer. B—Manually dissociated hESCs into smaller clumps and induced to form embryoid bodies (EBs) in non-adherent cell culture conditions. C—Morphology of first neural rosettes observed at days 4–12 after plating of EBs. D—Manually separated neural rosettes, dissociated into smaller pieces and transferred to new poly-l-ornithine/laminin-coated cell culture dishes. Upon adhesion, dissected clumps of rosettes began to generate new groups of rosettes (termed “R1”). E, F—Newly enriched population of neural rosettes, both fully reformed (E) and partially reformed (F), with a very small number of contaminating cells termed as “R2”. G—Independent “clone-like populations” of NSCs visible outside of rosettes-like structures. H, I—Manually isolated single “clone-like population” of NSCs and re-plated into 24 wells plate. J, K, L—Established self-renewing population of clonal morphology NSCs, further referred to as CoMo-NSCs at low density (J), high density (K) and high magnification (L). (scale bars: A 250 μm; B, C 500 μm; D–G 250 μm; H, I 150 μm; J, K 250 μm; L 100 μm). (JPG 2540 kb) [file 13287_2019_1163_MOESM4_ESM.jpg]

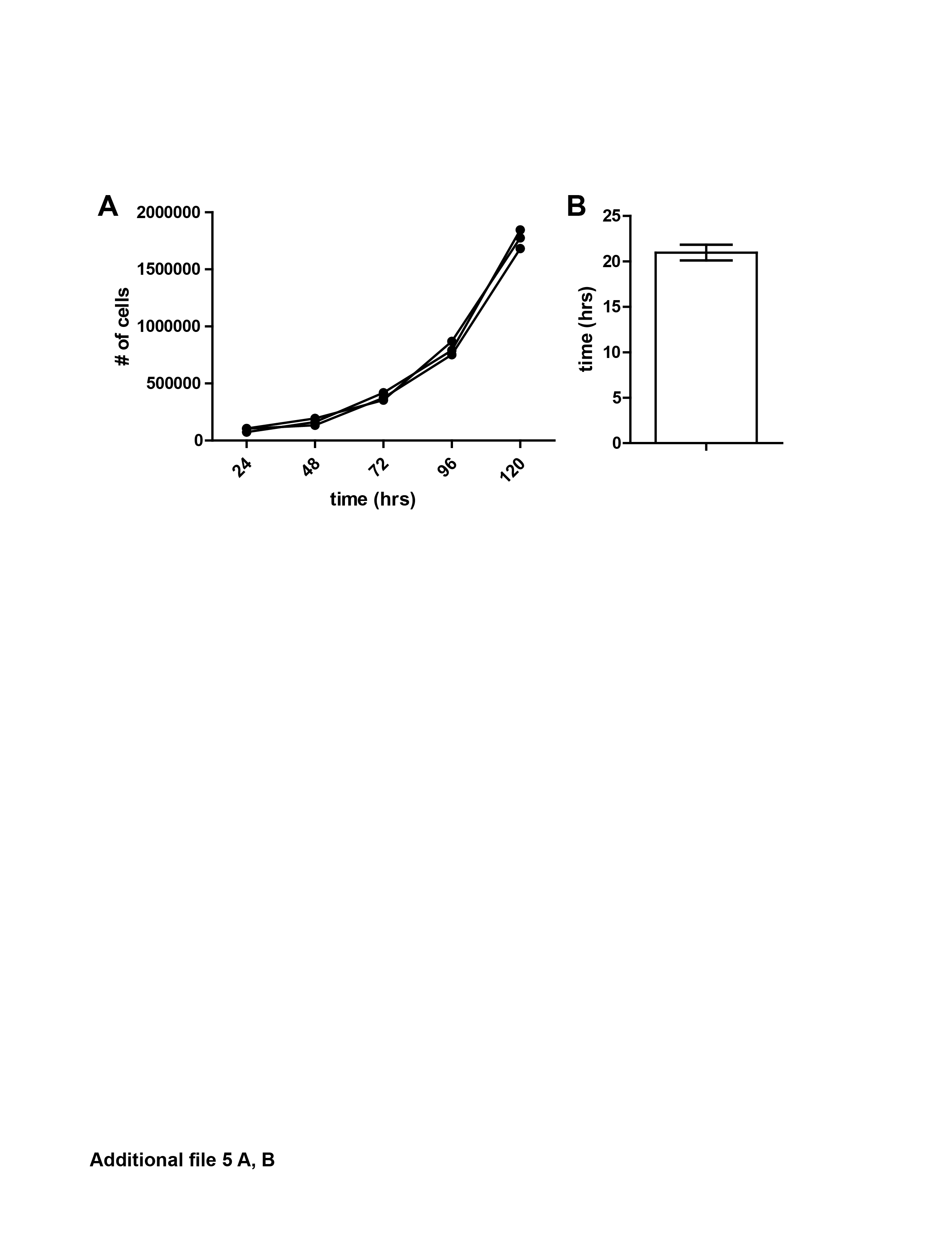

Supplement: Supplementary file 5 — Growth curve and doubling time of CoMo-NSCs. A—Growth curve from three independent cell lines of established CoMo-NSCs. B—Average doubling time of 20.96 h (± 1.51) was calculated using formula DT = t/3.3*log b/B between day 2 and day 4 (during the exponential phase of cell growth). DT = doubling time, t = time in minutes, b = number of cells at the end time point, B = number of cells at the first time point. (JPG 247 kb) [file 13287_2019_1163_MOESM5_ESM.jpg]

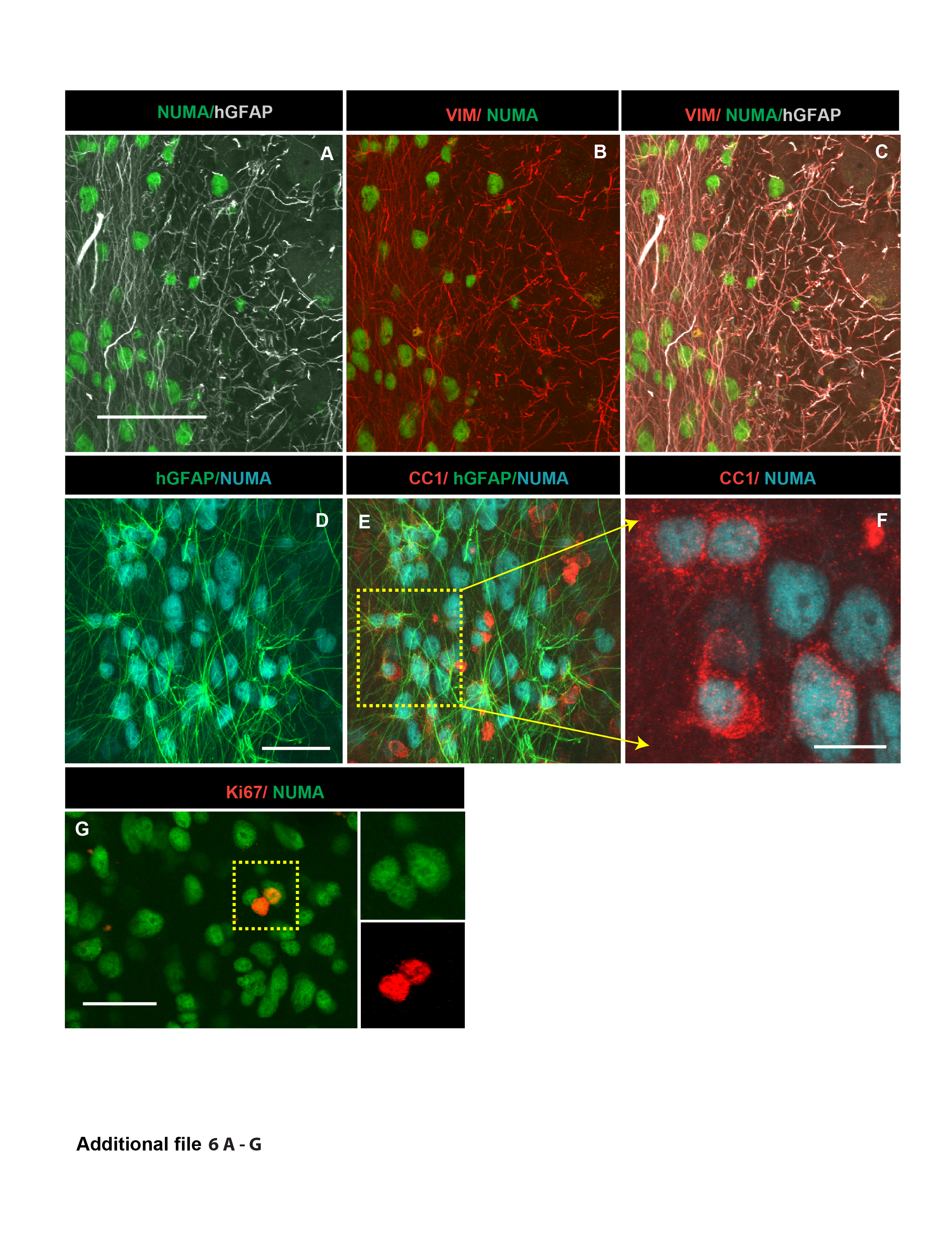

Supplement: Supplementary file 6 — Spinally grafted clonal NSCs give rise to mature astrocyte and oligodendrocytes in the immunodeficient rat at 6 months post-grafting. A, B, C—A high-density network of human-specific GFAP+ processes in the areas of hNUMA+ human grafts can be seen. D, E, F—In the same areas a subpopulation of hNUMA+ grafted cells expressed a mature oligodendrocyte marker CC1. G—Double staining with hNUMA and Ki67 antibody showed the only occasional presence of mitotically active grafted cells. (scale bars: A 100 μm; D 80 μm; F 10 μm; G 50 μm). (JPG 4957 kb) [file 13287_2019_1163_MOESM6_ESM.jpg]

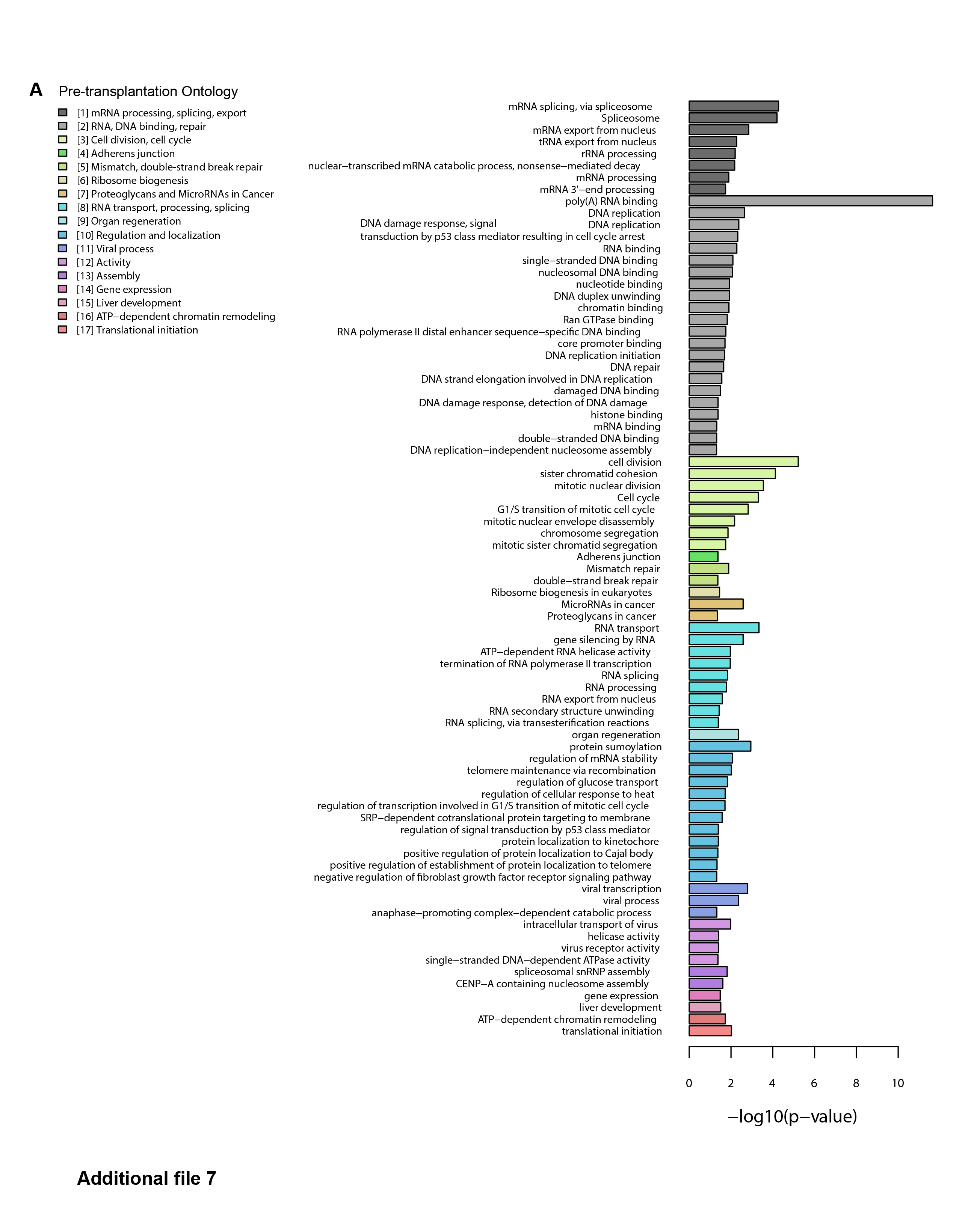

Supplement: Supplementary file 7 — Pre-transplantation gene ontology terms. A—Gene ontology terms overrepresented by genes enriched in the CoMo-NSCs pre-transplantation. (JPG 1072 kb) [file 13287_2019_1163_MOESM7_ESM.jpg]

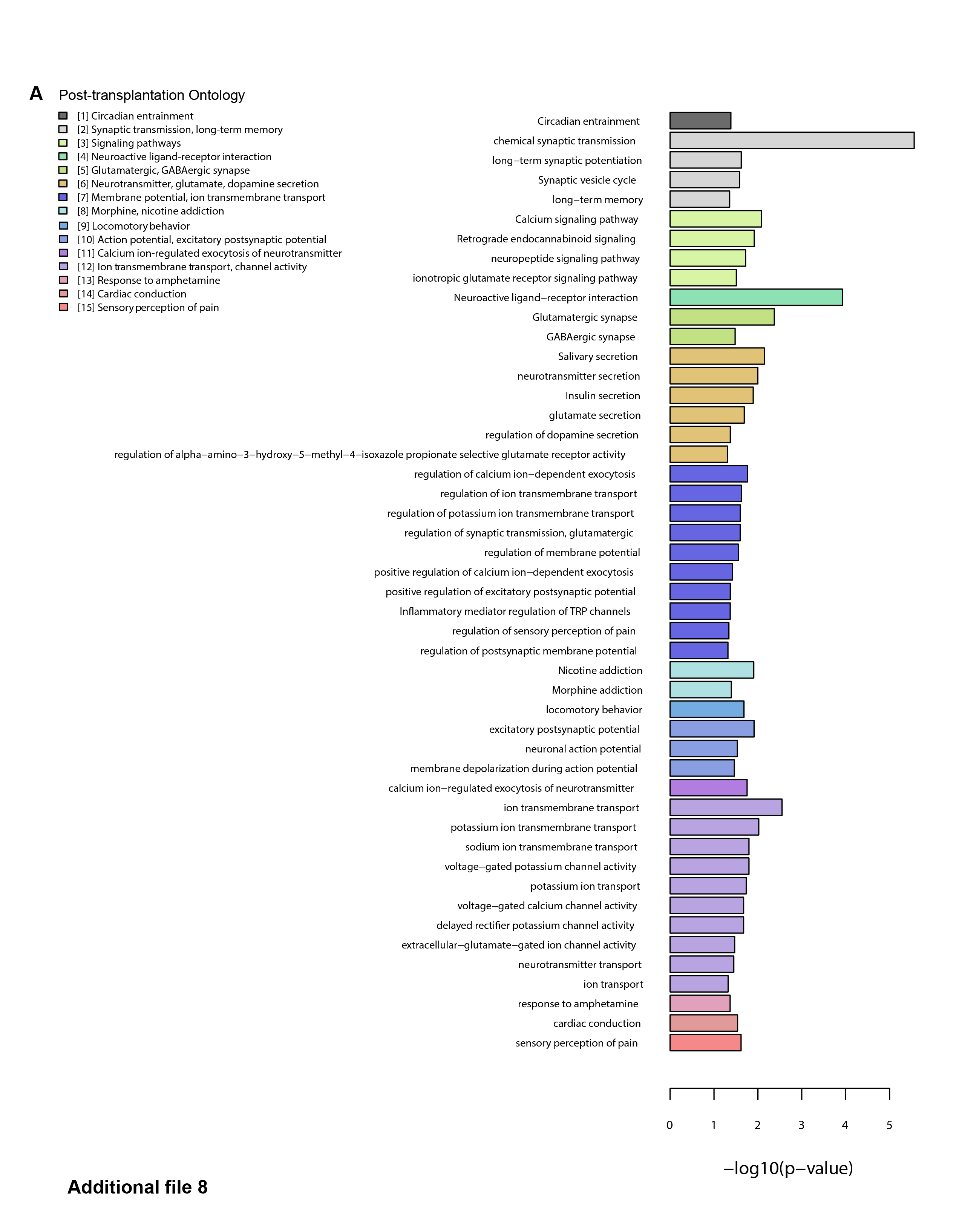

Supplement: Supplementary file 8 — Post-transplantation gene ontology terms. A—Gene ontology terms overrepresented by genes enriched in the CoMo-NSCs post-transplantation. (JPG 902 kb) [file 13287_2019_1163_MOESM8_ESM.jpg]

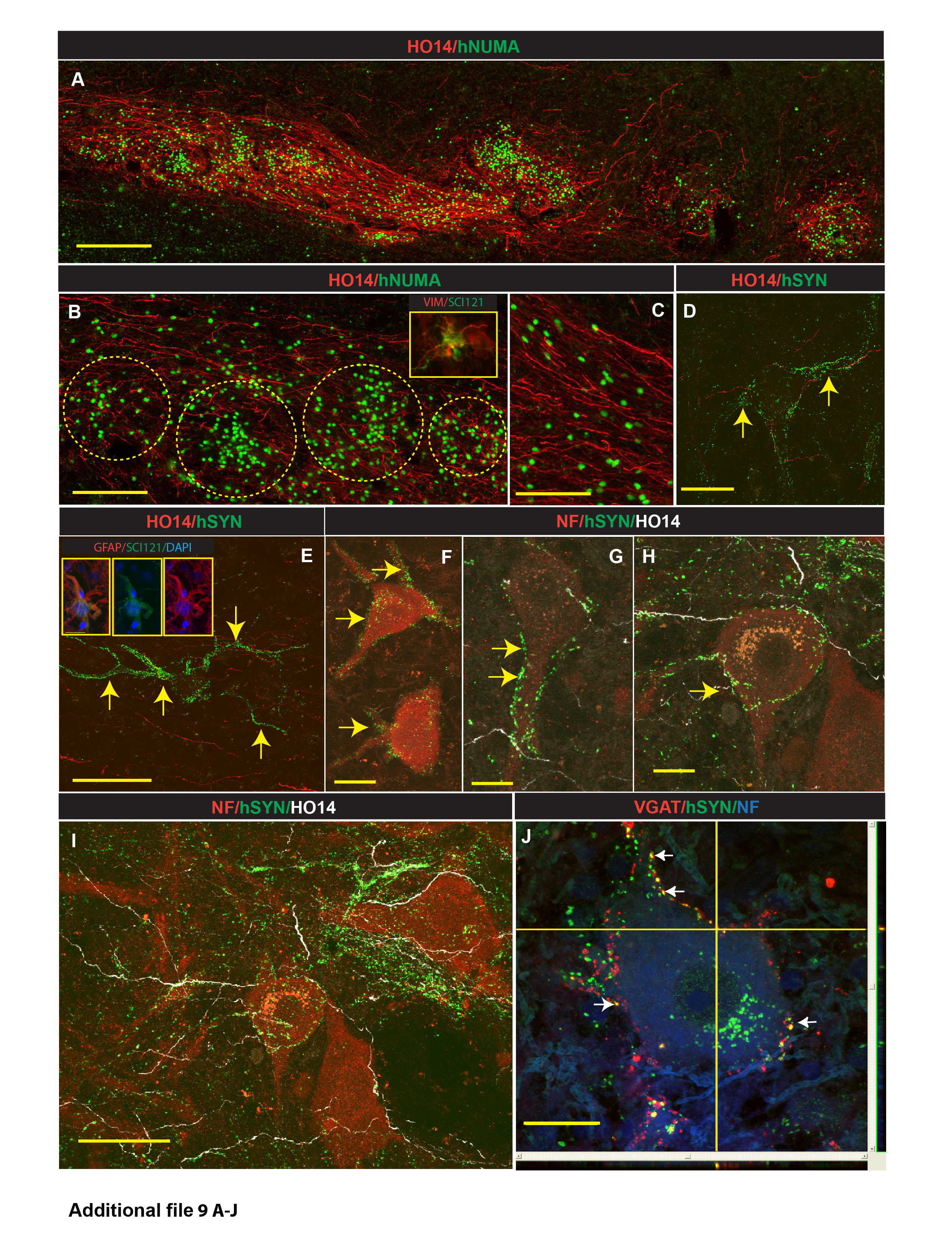

Supplement: Supplementary file 9 — Spinally grafted CoMo-NSCs-derived neurons show a long-term engraftment, no tumor formation and extensive axonal sprouting in adult pig with previous spinal injury. A total of 20 injections of NSCs were injected bilaterally above and below spinal injury epicenter (L2–L3 segments) in chronic spinally injured adult minipigs. The presence of grafted NSCs was analyzed at 3 months after cell grafting. A, B, C—Multiple clusters of hNUMA+ grafted cells (green signal) can be identified in horizontally cut section taken from cell-grafted region. In the same areas a high density of grafted neuron-derived axons (HO14-red signal) can be seen. D, E, F, G, H, I—Staining with human-specific synaptophysin antibody (green signal) showed a high density of hSYN puncta on the host NF+ neurons. Numerous grafted neurons-derived axons (HO14; white) in the vicinity of medium-sized and large host neurons can also be seen. Only few GFAP+ grafted astrocytes (colocalizing with pan-human SCI121 immunoreactivity) were seen (E; insert). J—Triple staining with human-specific synaptophysin antibody, VGAT (vesicular GABA transporter) and NF showed numerous double hSYN/VGAT-stained puncta on the membranes of large neurons of the host (white arrows). (scale bars: A 500 μm; B 100 μm; C 50 μm; D 20 μm; E 30 μm; F 20 μm; G 10 μm; H 10 μm; I 20 μm; J 5 μm) (JPG 8408 kb) [file 13287_2019_1163_MOESM9_ESM.jpg]
